# Rare Long‐Term Data Reveal the Seasonal Dietary Plasticity of Mandrills (*Mandrillus sphinx*) in Response to Fruiting Tree Phenology

**DOI:** 10.1002/ajp.70012

**Published:** 2025-03-17

**Authors:** Joshua Bauld, David Lehmann, Luc F. Bussière, Emma R. Bush, Edmond Dimoto, Jean‐Thoussaint Dikangadissi, Tharcisse Ukizintambara, Elizabeth C. White, Jason Newton, Isabel L. Jones, Lee J. T. White, Ruth Musgrave, Katharine A. Abernethy

**Affiliations:** ^1^ Biological and Environmental Sciences University of Stirling Stirling UK; ^2^ Agence Nationale des Parcs Nationaux (ANPN) Libreville Gabon; ^3^ Biology and Environmental Sciences and Gothenburg Global Biodiversity Centre University of Gothenburg Gothenburg Sweden; ^4^ Royal Botanic Garden Edinburgh Edinburgh UK; ^5^ BirdLife International Dakar Senegal; ^6^ Environment Programme World Conservation Monitoring Centre (UNEP‐WCMC) Cambridge UK; ^7^ National Environmental Isotope Facility Scottish Universities Environmental Research Centre East Kilbride UK; ^8^ Institut de Recherche en Ecologie Tropicale, CENAREST Libreville Gabon; ^9^ Wildlife Conservation Society Gabon Libreville Gabon

**Keywords:** fallback foods, *Mandrillus*, *Mandrillus sphinx*, nutrition, phenology

## Abstract

Understanding primate dietary plasticity provides insights into trait evolution and resilience to environmental change. Here, we investigate the feeding ecology of mandrills (*Mandrillus sphinx*), a species that forms groups of close to 1000 individuals, which presumably impacts feeding ecology by creating exceptionally high feeding competition. Mandrills are also threatened by habitat loss and climate change, and a full understanding of their dietary plasticity is essential to ongoing conservation efforts. Evidence suggests that mandrills are generalist feeders and consume a wide variety of resources to compensate for shortfalls in fruit availability. However, a lack of long‐term data on fruit production within the mandrill geographic range means that it is unknown whether the flexible feeding strategies observed previously are stable over multiple years. We combined two rare data sets comprising 8 years of fecal collection and fruit availability to assess the dietary flexibility of mandrills in Lopé National Park, Gabon. We found fruit to be the most frequently consumed resource and fruit consumption covaried positively with fruit availability, peaking during periods of fruit abundance. Mandrill dietary diversity increased during periods of fruit scarcity, through greater consumption of animal prey, leaves, seeds, and other plant fibers. These results demonstrate that mandrills are primarily frugivorous, but that they are also highly flexible feeders, able to respond to temporal variation in fruit production over several annual cycles. In addition, we found that mandrills varied in the extent to which they preferred different fruit taxa. Lipid‐rich oil palm (*Elaeis guineensis*) fruits were by far the most frequently consumed resource and may constitute a staple resource for mandrills in the study site. Our multiyear study provides robust evidence for generalist feeding behavior by mandrills, which may be driven by extreme group sizes or past environmental fluctuations and provide resilience to future environmental change.

## Summary

- Across 8 years of data collection, mandrills (*Mandrillus sphinx*) exhibited generalist feeding strategies, characterized by high fruit consumption during wet seasons and fallback food consumption during dry seasons.
- Mandrill displayed preferences for particular fruit genera, which is likely driven by fruit macronutrient contents. In particular, lipid rich oil palm (*Elaeis guineensis*) fruit are a staple resource to mandrills in our study site.
- Diet breadth was maintained across seasons and several years, suggesting that generalist feeding behavior is consistent within and between annual cycles. Major fallback foods consisted of animal prey, leaves, crushed seeds, non‐fruit fibers.

## 1 Introduction

Diet has long been a focus of primatological research (Lambert and Rothman 2015) because of its relevance to topics such as species coexistence (Houle et al. 2006), space use (Hanya 2004; Zhang et al. 2021), life histories (Borries et al. 2011), and morphological trait evolution (Regan et al. 2001; Onstein et al. 2020). Furthermore, habitat destruction and climate change are intense conservation challenges for primates, many species of which are at high risk of extinction (Pacifici et al. 2017; Bernard and Marshall 2020). Investigating dietary flexibility could provide insights about primates' resilience to change and identify the most (and least) effective conservation strategies (Harcourt et al. 2002; Nowak and Lee 2013). A greater understanding of wild primate diets may also inform the ongoing debate about optimal human nutrition (Milton 2000, 2003; Alt et al. 2022).

Long‐term behavioral and habitat data are key to a full understanding of primate ecology and evolution (Chapman et al. 2017; Melin et al. 2020). In the case of diet, long‐term data facilitate inferences about feeding ecology that account for interannual changes in variables such as food availability (Chapman et al. 2002; Zhou et al. 2009; Erhart et al. 2018). In this study, we use rare multiyear data sets on diet and fruit production to investigate the feeding ecology of mandrills (*Mandrillus sphinx*), by analyzing seasonal changes in diet across eight annual cycles. We focus on mandrills because their large social groups (Abernethy et al. 2002) and extreme sexual dimorphism (Setchell 2016) may greatly impact dietary plasticity at the individual and group levels.

One of the major influences on primate diets is seasonal variation in food availability (Van Schaik et al. 1993; Peres 1994; Tuyisingize et al. 2022). The seasonal timing of biological events, such as fruit production, is termed phenology (Leith 1974), which in tropical trees is influenced by temperature and rainfall (Reich 1995; Mendoza et al. 2017; Potts et al. 2020). Frugivorous primates rely on fruit as their principal food source and, as a result, typically exhibit seasonal dietary variation in response to phenological cycles of fruit production (Guo et al. 2007; Chancellor et al. 2012; Butt et al. 2015; DeLuycker 2021). The taxonomic diversity and abundance of fruit may, however, vary between years because the fruiting phenology of different tree species can follow annual, sub‐, or supra‐annual cycles (Bush et al. 2017; Adamescu et al. 2018). As a consequence, our primary goal was to describe average seasonal changes in mandrill food selection across multiple years, that may have varied in exactly which fruit species were available.

Fluctuations in fruit abundance result in periods of scarcity through the year, and during these times, frugivorous primates are expected to exhibit dietary and behavioral flexibility to make up for any nutritional shortfalls (Tutin et al. 1991). Possibilities include switching to other food types, such as leaves or invertebrates; feeding on fruits, which are more consistently available, but higher in fiber or defensive compounds(Hill 1997; McConkey et al. 2002; Clink et al. 2017); foraging over larger areas (Nagy‐Reis and Setz 2017); or reducing group or party size, to exploit smaller food patches (Tutin and Fernandez 1993). “Preferred” food types may, therefore, be identified as those for which consumption covaries positively with availability (Leighton 1993). In contrast, “fallback” foods can be considered as alternative food types, the consumption of which covaries negatively with the consumption of preferred resources (Marshall and Wrangham 2007). This switching to alternative resources may furthermore be accompanied by an increase in dietary diversity, as primates attempt to make up the nutritional shortfalls of alternative food sources (Lambert and Rothman 2015).

In addition to assessing which fallback foods are consumed, knowledge of which fruits are selected during periods of abundance is also necessary for understanding frugivore feeding strategies (Leighton 1993; Doran‐Sheehy et al. 2009). Fruits that are consumed in greater amounts as a function of availability may be considered “preferred” and those consumed less as a function of availability may be classified as “avoided” (Russo et al. 2005). A robust understanding of how primate diets vary in response to phenology is an important first step to understanding their feeding strategies (Chapman et al. 2002). For example, some foods may be targeted because of their macronutrient contents (Conklin‐Brittain et al. 1998) and some may be avoided due to containing plant defensive compounds (Masette et al. 2015).

Primate food selection may also be affected by within‐ and between‐species resource competition. The formation of social groups results in an aggregation of animals in space that then produces competition for food resources, the intensity of which is moderated by group size and the distribution of resources in space (Wrangham 1980; Van Schaik et al. 1989; Sterck et al. 1997). Feeding competition may be indirect, when individuals (or groups) compete to arrive first at a resource (Miller et al. 2020), or direct, if dominant individuals (or groups) exclude others from a resource (Scarry 2013). In multispecies assemblages, both indirect and direct competition may also occur between species (Wahungu 1998; Sushma and Singh 2006; Ledogar et al. 2013). The results of within‐ and between‐species feeding competition is that, even when food availability is high, preferred resources may be inaccessible to subordinate individuals, groups, or species (Houle et al. 2010; Houle and Wrangham 2021). Feeding competition may, therefore, act as an additional source of variation in food selection, alongside seasonal changes in food availability.

Mandrills are a particularly interesting species in which to study the relationship between tree phenology and diet because they exhibit several exceptional traits relevant to food selection. Though classified as frugivores, mandrills consume a remarkably wide range of foods, including vertebrate and freshwater prey (Jouventin 1975; Hoshino 1985; Harrison 1988; Norris 1988; Rogers et al. 1996). Mandrill groups, termed “hordes” due to their fission‐fusion social structure (White 2007), are highly flexible and variable in size (Hoshino et al. 1984; Hongo 2014; Brockmeyer et al. 2015), but can be exceptionally large, numbering 620 (range = 340–845), on average, at the site of the present study (Abernethy et al. 2002). Such large group sizes likely result in high levels of within‐group feeding competition and rapid rates of patch depletion. Consequently, mandrills also occupy some of the largest total home ranges documented in any wild primate (118 km^2^, 46 km^2^ of forest) (White et al. 2010) and may travel up to 10 km per day (White 2007; Hongo et al. 2022). These socioecological traits are coupled with the most extreme size dimorphism seen in primates and extravagant facial adornments on male animals (Darwin 1871; Setchell 2016). Furthermore, the geographic range of mandrills overlaps with those of numerous other primate species and high biomass frugivores, such as forest elephants (*Loxodonta cyclotis*) and red river hogs (*Potamochoerus porcus*) (Tutin et al. 1997). Individual mandrills must, therefore, cope with high levels of feeding competition to consume a diet that provides sufficient energy to sustain extensive travel, with nutritional requirements also potentially differing between sexes due to extreme dimorphism.

Periods of fruit scarcity may hamper the ability of individual mandrills to consume sufficient resources. Previous investigations have documented that when fruit availability is low, mandrill feeds on a variety of fallback foods, with a consequent increase in dietary diversity (Nsi Akoue et al. 2017; Hongo et al. 2018). Tree phenological cycles and fruit availability may vary between years, however, and so whether this feeding strategy remains consistent over several annual cycles is an open question, unanswered primarily due to a lack of long‐term data on either tree phenology or mandrill diets (White 2007; Hongo et al. 2018).

In this study, we investigate mandrill feeding strategies using a rare 8‐year data set on mandrill diets obtained from fecal samples gathered over an 8‐year period in Lopé National Park, Gabon. We analyze these data in conjunction with a long‐term (1986‐present) tree phenology data set, the longest continuous data set of its kind in Africa (Adamescu et al. 2018) and the only one within the known geographic range of mandrills. The Lopé phenology data set indicates that fruit abundance varies seasonally, with fruit production peaking in the two wet seasons (February–May and September–November) and falling in the two dry seasons (June–August and December–January) (Bush et al. 2017). This fluctuation in fruit availability has previously been observed to influence the diets of primates living within the park (White et al. 1994; Tutin et al. 1997). However, the exact timing and duration of each season, as well as the total amount of fruit produced, may vary between years (Tutin et al. 1991; Bush, Whytock, et al. 2020). By combining the fecal and phenology data sets, we are therefore able to build upon prior investigations with an analysis of mandrill feeding strategies that account for long‐term variation in tree phenology and fruit availability.

To investigate mandrill diets, we analyzed 4024 fecal samples collected between September 1996 and October 2004, from which we recorded the major food types consumed and identified plant tissues to species level, where possible. Using these data, we first set out to describe our study horde's diet in terms of preferred food types, use of fallback foods, and seasonal changes in food type diversity. Mandrills also appear to prefer some fruits over others during periods of abundance (White 2007; Nsi Akoue et al. 2017). We, therefore, also examined whether particular fruits were consumed more frequently as a function of availability, suggesting they are preferred foods, and explored whether preferences may exist in terms of maximum tree height and nutritional contents. We analyzed the impact of maximum tree height on the frequency at which fruit genera were consumed because a negative association could suggest that mandrills avoid foraging in tall trees or are excluded from the tops of trees by other frugivorous species. Similarly, we examined the relationship between nutritional contents (data from Elizabeth Rogers et al. 1990) and consumption frequency to try and elucidate the mechanisms driving mandrill food selection.

## 2 Methods

### 2.1 Study Site

**Figure 1:**
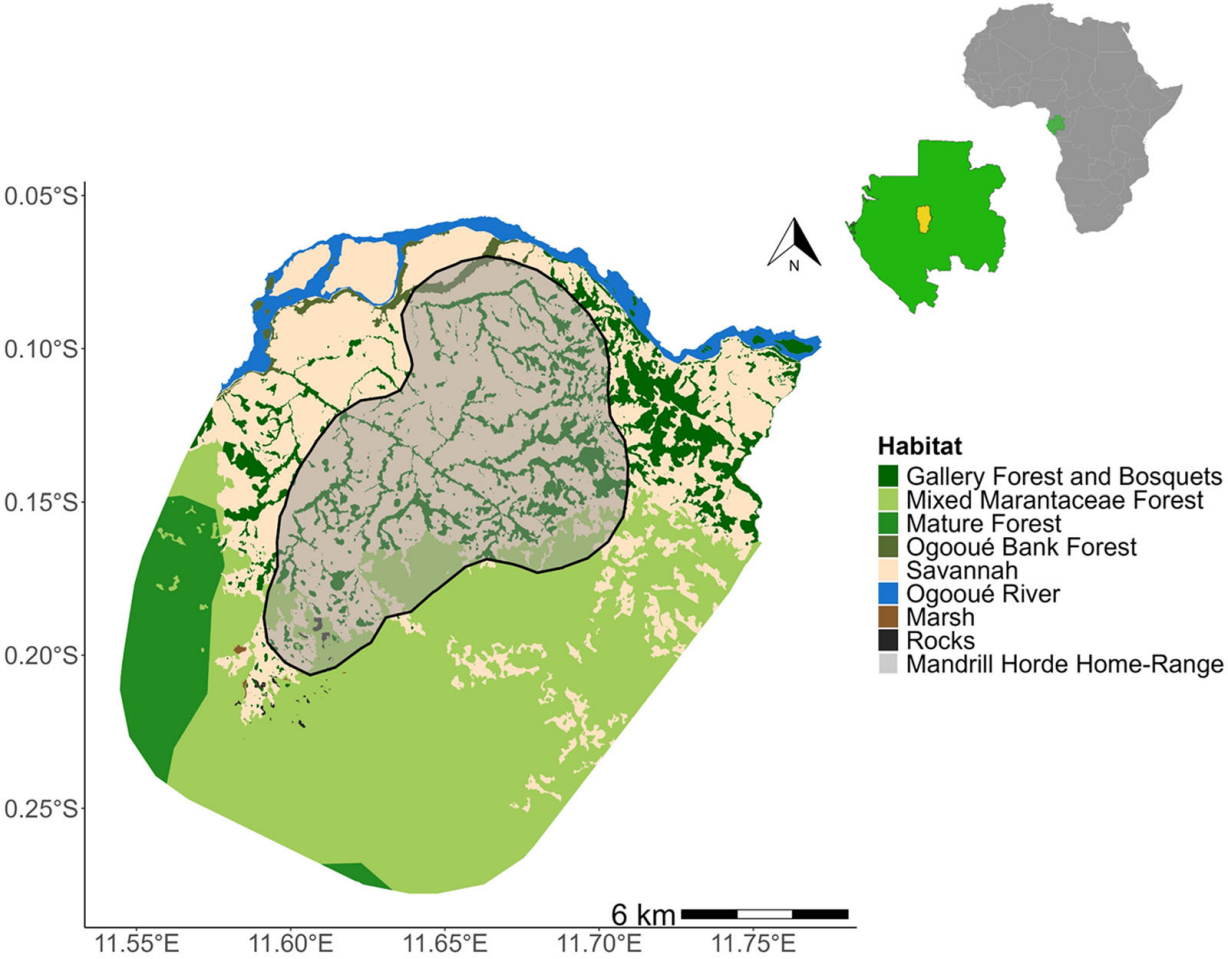
The location of Lopé National Park in Gabon, Central Africa, and the distribution of habitats in the study site, with the home‐range (95% fixed kernel contours) of the focal horde overlaid (data from White et al. 2010).

Our study was carried out in Lopé National Park, located in Gabon (−0.2 N, 11.6 E), Central Africa (Figure 1). Lopé National Park covers an area of 4964 km^2^ which is mostly old‐growth Guineo‐Congolian evergreen tropical rainforest but contains a dynamic forest‐savanna mosaic landscape covering approximately 10% of its northern area (Figure 1). This mix of savanna and gallery forests is bordered to the north by the Ogooué (the second largest river in the Congo‐Ogooué basin). Our 182 km^2^ study area, based on the area used by mandrills in White et al. (2010), is situated within the forest‐savanna matrix and adjacent continuous forest (Figure 1). The dominant type of vegetation at the continuous forest edge in this area is “Marantaceae forest,” which is a young secondary forest with a dense herbaceous understory dominated by plants of the families Marantaceae and Zingiberaceae. Gallery forest fragments that extend out from the main continuous forest block into the savanna typically form along small riverbeds and have sparse ground vegetation of lianas and small shrubs. Plant species assemblages and compositions differ between gallery forests, forest fragments and continuous forests (see White 1994; White and Abernethy 1997; Tutin et al. 1997, Léal 2004; Ukizintambara et al. 2007; White 2007). Notably, the gallery forests have a history of anthropization over several hundred years, which included the planting of oil palms (*Elaeis guineensis*) (Maley and Chepstow‐Lusty 2001; Bostoen et al. 2013). Lopé National Park receives on average 1466 ± 201 mm of rain per year, which falls within two distinct time‐windows: the long rainy season runs from February to May, and the shorter rainy season from September to November. The short dry season takes place from December to January, while the longer dry season occurs from June to August (White 1994; Bush, Jeffery, et al. 2020).

### 2.2 Study Population

Our study population consisted of a mandrill horde, usually numbering between 600 and 800 individuals, though temporary extreme counts of as many as 1350 individuals have been recorded in the past when two hordes meet (Abernethy et al. 1997). The group exhibits fission–fusion dynamics, particularly during periods of low food availability (White 2007), meaning that the number of individuals varied considerably throughout our 8‐year study period (Abernethy et al. 2002). Over the period January 2003 to October 2004, the tracking of radio‐collared females in the study population indicated that the horde was split into subgroups for at least 69% of days (and potentially > 90% of the time if sightings of subgroups without radio‐collared females but within the core home range were considered). Film recordings of the study population over this period (*n* = 27) ranged from 50 to 717 individuals (median = 357 individuals) (White 2007). Consequently, the level of within‐group feeding competition may have varied throughout the sampling period, potentially impacting food selection. In addition, adult males often leave the horde outside of the mating season, but even during mating, the proportion of adult and sub‐adult males has never been observed to exceed 12% (Abernethy et al. 2002), and so our fecal samples were mostly sourced from adult females and juveniles of both sexes. Our analysis of mandrill diets thus largely corresponds to permanent horde members and fewer adult males.

### 2.3 Fecal Collection and Analysis

To investigate seasonal changes in mandrill food selection, we collected 4024 fecal samples over 8 years and 2 months between September 1996 and October 2004. For all feces collections, the mandrill group being sampled was identified as being all or part of the two hordes known to use the study area, using radiotelemetry to identify the presence or absence of collared individuals (see Abernethy et al. 2002; White 2007). Only fresh dung (10 min to 5 h old) was collected to minimize the loss of material to fecal predators. When a dung pile was found, the entire fecal pellet was collected into a plastic ziplock bag and conserved at ambient temperature until analysis. Collections were made weekly on a predetermined day to prevent bias to collections in more accessible habitats and to ensure an even representation throughout the year. When logistical constraints prevented collections from being made on the determined day of the week, efforts were continued to contact the group and samples were obtained as soon as possible after this date.

Identification of dietary items was made within 3 days of collection to ensure that constituent parts were recognizable and had not been degraded. Analysis of constituent parts followed the protocol established at Station d'étude des Gorilles et des Chimpanzés by Tutin and Fernandez (1993) for analysis of ape dung. We used 1 mm mesh brass soil sieves to separate the matrix with running water and retain identifiable undigested components. Cleaned dung components were identified to plant parts and species as far as possible, using the herbarium and reference collections of seeds curated at SEGC (White and Abernethy 1997). Where species level identification was not possible, plant genus, plant family or finally “component type,” for example, “unknown stems” or “plant fiber,” classifications were made. Animal and insect remains could rarely be classified beyond Order level (e.g., mammal, reptile, insect, mollusk), however, where possible, a more precise taxon was noted.

### 2.4 Descriptive Analyses of Food Types and Plant Taxa

To analyze seasonal changes in consumption of major food types, we classified identified foods into one of seven categories. In order of prevalence within mandrill fecal samples (see Section 3): the whole seeds, pulp, skins, and fibers of identified fruit species were pooled as “Fruit”; chitin from insects, hair, bone, and other vertebrate or invertebrate remains were grouped as “Animal Parts”; dicotyledonous leaves were categorized as “Dicot Leaves”; unidentified fibers or plant material, pieces of bark, twigs, and pieces of wood were termed “Other Fiber”; seeds that showed clear evidence of seed predation (rather than fruit consumption and seed dispersal) were marked as “Crushed Seeds”; pieces of mushroom were categorized as “Fungus”; and leaves from monocotyledonous plant species were clustered as “Monocot Leaves.”

Alongside seasonal changes in consumption of each food type, we also aimed to analyze seasonal changes in diet breadth. Here, we define “diet breadth” as the count of different food types in a fecal sample. We then define “fruit breadth” as the count of distinct fruit genera present in a fecal sample. We can, therefore, compare changes in diet breadth and fruit breadth throughout the year, to describe temporal variation in the contribution of fallback (i.e., non‐fruit) foods to mandrill diet breadth. To classify different plant taxa within the fruit breadth variable, we grouped different tree species to the genus or family level because species‐level identifications were not always possible. For example, for *Uapaca* sp., there are three species, *U. heudeleotti*, *U. paludosa*, and *U. guineensis*, present in the study area and their seeds are difficult to reliably tell apart, especially when damaged. This grouping resulted in a loss of some dietary resolution but allowed us to maximize the sample size for characterizing the phenology of food items in mandrill dung.

Throughout our analyses of seasonal variation in food selection and diet breadth we focus on the presence versus absence of food items, rather than quantifying the amount of each in fecal samples. This is partly because comparisons of quantities across items is difficult (e.g., for fruits with dramatically different seed sizes), but also because fecal pellets represent only the undigested fraction of food and could therefore easily misrepresent food selection or underestimate unidentifiable remains (Tutin and Fernandez 1993).

### 2.5 Fruit Availability

To facilitate comparisons between fruit consumption and fruit availability and thus identify preferred or avoided fruit taxa, we calculated fruit availability using data on tree fruiting phenology, tree abundance, and tree size. Tree phenology has been monitored in Lopé National Park since 1986 to present, with phenology circuits and tree surveys conducted throughout our 182 km^2^ study area (Bush et al. 2017). Field researchers use binoculars to record canopy cover of immature and mature fruits on a monthly basis, rating coverage on a nine‐point scale from 0 for no coverage to 4 for complete coverage, using increments of 0.5 (Bush et al. 2017). Stem density (stems ha^−1^) and mean diameter at breast height (cm) (converted to radius) measures were taken from existing census data for each of the four major forest types in the study area (White 1994; Cardoso et al. 2020). Phenology data were available for 30 species, in 23 of the 53 genera found in mandrill feces, and we obtained a subset of the data available for these species; that matched the date range of the fecal sampling. We then calculated monthly fruit availability scores (FAS) for each species, following the method of Cardoso et al. (2020):

$$\text{FAS}=p\times c\times d\times r^{2},$$

where *p* is the proportion of a species bearing fruit in any given month; *c* is the mean proportion of the canopy of each species covered by mature fruit; *d* is the mean stem density of a species across all census plots in a habitat type; and *r* is the mean radius at breast height of a species across all census plots in a habitat type. Monthly FAS were calculated for each species in each habitat type and then multiplied by the total area of each habitat within the 182 km^2^ study area. FAS for each species in each habitat were then summed to produce a measure of the total monthly availability of each fruit species across our study site. Thus, we produced measures of fruit availability that could be compared directly to the frequency at which fruiting taxa appeared in mandrill fecal samples, which were also collected on a monthly basis. In cases where FAS were calculated for multiple species in the same genus, we summed the FAS for all species in a genus, so that our measure of fruit availability matched the taxonomic resolution of our fecal data set.

### 2.6 Statistical Analyses

We first broadly described the occurrence of each food type in the diet of our focal horde by calculating the proportion of all fecal samples in which each food type was present and produced 95% confidence intervals around these proportions with binomial exact tests, using the total sample of binary observations of presence and absence of each foot type. To quantify how the probability of occurrence of major food types varied through time we fitted generalized additive mixed models (GAMMs) with a logit‐link function and binomial error structure using the “mgcv” package v1.3.89 (Wood 2017) in R v4.1.3 (R Core Team 2022). GAMM models make no assumptions about the functional form of a curve but rather allow us to estimate the curvature best supported by the data. Fecal sampling events occurred across all four seasons (long wet, long dry, short wet, short dry), but to raise temporal resolution, we focused instead on the day of the year on which each sample was collected (e.g., January 1st = Day 1). This allowed us to estimate changes in prevalence at finer temporal scales than those available when considering seasons as discrete periods. In the GAMMs for each food type (equivalent to Model G in Pedersen et al. (2019)), we included a smoothing term for day of year as a fixed factor to describe the effect of day of year on the binomial presence of each food type in the diet. Day of year was specified as a cyclic cubic regression term to ensure that the intercepts for early January and late December aligned. We also included random slopes for year as an additional smoother so that the curves describing the frequency at which each food type appeared in mandrill diets accounted for differences between years in mandrill feeding. Our model structure, therefore, tends to penalize differences between sampling years, in proportion to the difference between each year and all other years. Smoothing parameter estimation was conducted using the Un‐Biased Risk Estimator.

To analyze whether mandrills compensate for low fruit availability by consuming alternate resources, we modeled temporal changes in diet breadth and fruit breadth using Poisson GAMMs, with a log‐link function, including day of year as a fixed effect and random intercepts for year. We produced the diet breadth response variable by summing the number of items in a fecal sample belonging to the different food type categories used above (e.g., 1× fruit, 1× dicot leaf, 1× animal part, equals a diet breadth of three). We then produced the fruit breadth response variable by summing the number of different fruiting genera present in a fecal sample (e.g., 1× *Dialium* sp., 1× *Uapaca* sp. equals a fruit breadth of two). To test whether consumption of fallback foods was statistically associated with fruit consumption, which is not directly achieved by comparing the two GAMM models, we compared the proportion of fecal samples containing non‐fruit food types when fruit was present or absent, using Chi‐squared tests.

To assess evidence of preference by mandrills for certain fruit genera, we used a binomial generalized linear mixed model, with consumption of fruit as the response variable and scaled fruit availability (without mean centering) as the predictor variable. A positive relationship between fruit availability and consumption would suggest that mandrills consume more fruit when it is most available, whereas no association between fruit consumption and availability would imply that fruit is not a preferred resource of mandrills. Similarly, relative differences in the strength of relationships between the availability and consumption of different fruit genera would also imply differences in the extent to which they are preferred by mandrills. Therefore, we also included a random slope argument in this model, allowing the relationship between availability and consumption to differ between fruiting genera, interpreting steeper positive slopes as an indication of greater preference by mandrills. We then compared the random slope values for each genera to the mean effect of fruit availability on consumption, classifying the genera with values greater than average as preferred and those with values lower than average not preferred. We opted for the term “not preferred” in place of “avoided” as some genera were among the most frequently consumed, despite not being among the most preferred genera, suggesting the term “avoided” would be inappropriate. The goodness of fit of the model containing random slopes for each genus was compared to one containing random intercepts and a fixed effects‐only model using Likelihood Ratio Tests.

We next examined whether maximum tree height and fruit nutritional contents (protein, carbohydrates, fat, water, fiber, tannins, or phenols) were associated with the frequency at which different fruits were consumed. To do so, we ran separate generalized linear mixed models, with tree height or each nutritional trait as a continuous predictor variable, random intercepts for each fruit genus, and the binomial presence of each genus in mandrill feces as the response variable. We took maximum tree height data from the primary vegetation guide for Lopé National Park (White and Abernethy 1997) or online databases of plant traits and nutritional data from Elizabeth Rogers et al. (1990). For all models, we visually inspected diagnostic plots to ensure good model fit and adherence to model assumptions. We also explored the sensitivity of our GAMMs to the number of smoother knots, *k*, using the *gam.check* function. For a few models, the default number of smoothers suggested the possibility of underfitting. However, increasing *k* did not reveal patterns that altered our interpretation and so, for simplicity and consistency, we present fitted values for curves from models with *k* = 8 for fixed effects and *k* = 74 for the random slope effect of the year.

## 3 Results

We grouped the foods that mandrills consumed into seven categories: fruit, animal parts (including vertebrates and invertebrates), dicotyledonous leaves, monocotyledonous leaves, crushed seeds, fungi, other fibers (including non‐fruit fiber and wood). The mandrills consumed a minimum of 67 different plant species, which is a conservative estimate, as not all samples could be identified to species level, and many of the families and genera identified have multiple representatives within the horde's home range (Table SA1).

**Figure 2:**
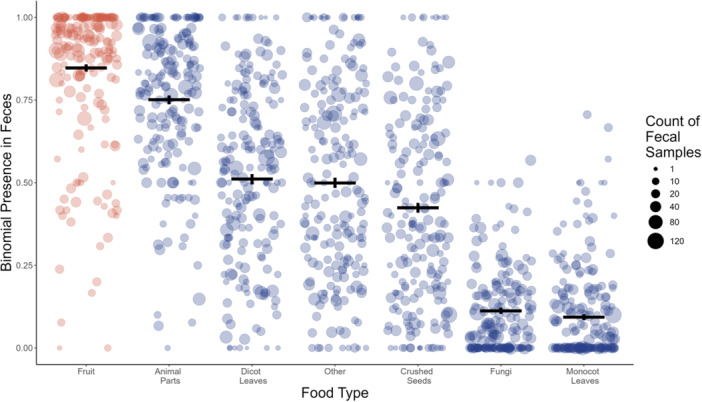
Consumption of major food types by the focal horde, across all fecal samples. Data points represent an individual day of the year on which feces were sampled. The position of points on the *y*‐axis indicates the proportion of fecal samples containing a given resource, averaged across all sampling years, and based on binomial presence versus absence. Point size represents the number of fecal samples collected on a given day of the year, summed across sampling years. Horizontal lines indicate the proportion of all fecal samples containing a given food type and vertical black lines indicate the 95% binomial confidence intervals around those proportions.

Fruit was the most frequently consumed food type, based on binomial presence versus absence, present in 84.8% (95% CI: 83.7–85.9) of fecal samples. The second most frequently consumed food type was animal parts (75.1%, 73.7–76.4), followed by dicotyledonous leaves (51.1%, 49.6–52.7), other fibers (50%, 48.4–51.5), crushed seeds (42.4%, 40.9–44), fungi (11.3%, 10.3–12.3), and monocotyledonous leaves (9.3%, 8.5–10.2) (Figure 2).

**Figure 3:**
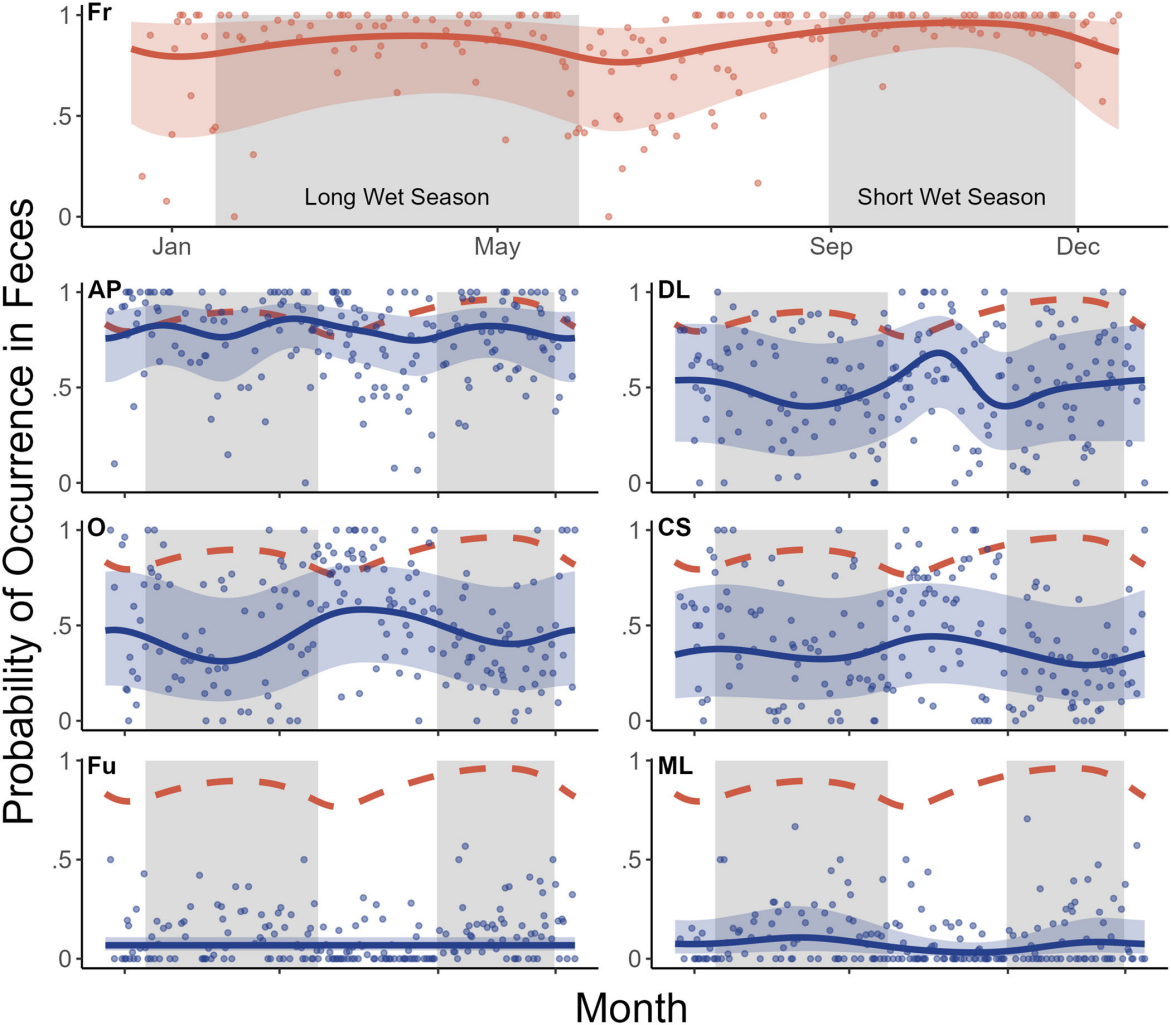
Generalized additive model examining the effect of day of year on consumption of major mandrill food types (Fr = fruit, DL = dicot leaves, Fu = fungi, O = other, ML = monocot leaves, CS = crushed seeds, AP = animal parts). Solid lines indicate the predicted binomial probability of a given food type being found in a fecal sample, on a given day of the year. Ribbons indicate the 95% confidence interval around the predicted binomial probability. The model output is displayed over raw data to visualize the relationship between consumption and day of year. Each data point represents one sampling day, and their position on the *y*‐axis indicates the proportion of fecal samples on that day containing a given food type. Dark and light shaded areas are indicative of wet and dry seasons, respectively. Dashed red lines illustrate the predicted binomial probability of a fecal sample containing fruit, for comparison to other food types.

Generalized additive models indicated that the consumption frequency of six out of seven mandrill food types was associated with the day of year (Figure 3). Fruit consumption peaked during the two wet seasons (Figure 3: Fr; Table SA2), whereas some alternative food types showed inverse trends compared to fruit, with crushed seeds, dicot leaves, and other fibers all exhibiting two peaks in the dry seasons, when fruit consumption was lowest (Figure 3: DL, O, CS; Tables SA4–SA6). Consumption of animal parts appeared somewhat independent of fruit consumption. Peaks in animal consumption were present at the end of the long wet season when fruit consumption began to decline, but also at the beginning of the long wet season, when fruit consumption was increasing, as well as during the short wet season, when fruit consumption was relatively high (Figure 3: AP; Table SA3). We found no evidence of an association between the day of year and the probability of fungi consumption, as indicated by an entirely flat curve, which reflects the lack of any seasonal pattern in fungi consumption visible in the raw data (Figure 3: Fu; Table SA7). Consumption of monocot leaves had two small peaks in the wet seasons (Figure 3: ML; Table SA8).

The inclusion of random smooths to account for variation between years in the shape of relationships between the day of year and the consumption probability of each food type resulted in very wide confidence intervals around the mean probabilities across years (Figure 3). The reason for these wide confidence intervals was substantial interannual variation in consumption of all food types (Figures SA1:SA9). For example, fruit consumption was high in the wet seasons and low in the dry seasons of 2002 but high in the dry seasons of 1997 and 2001 (Figure SA1). Similarly, consumption of other fibers was high in the dry seasons and low in the wet seasons of 2000, but low in the dry seasons and high in the wet seasons of 2001 (Figure SA3).

**Figure 4:**
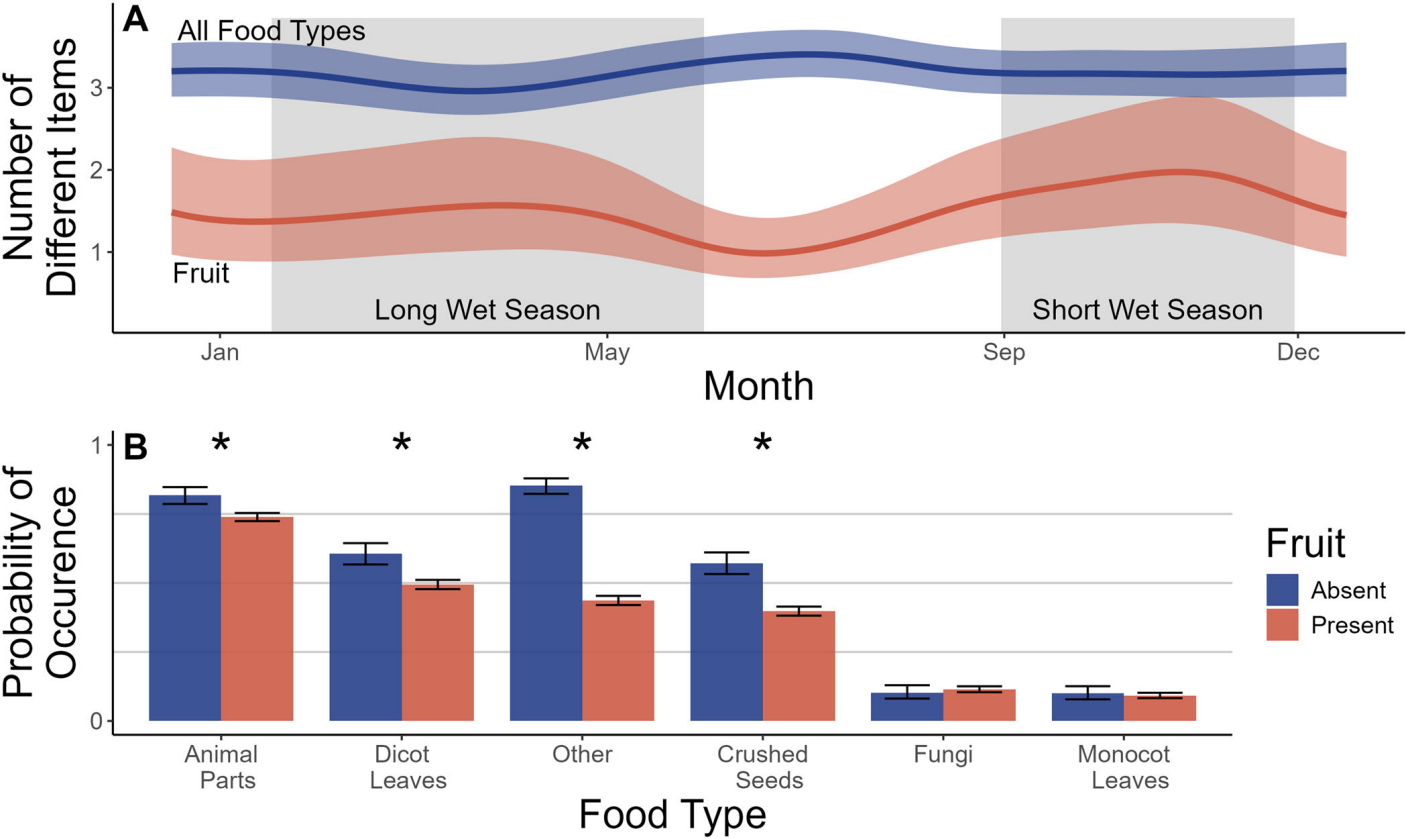
(A) Generalized additive models illustrating the relationship between day of year and overall dietary breadth (sum of different food types) and dietary fruit breadth (sum of fruit genera). Lines indicate the predicted binomial probability of a given food type being found in a fecal sample, on a given day of the year (Day 1 = January 1st). Ribbons indicate the 95% confidence interval around the predicted binomial probability. (B) The binomial presence of major food groups in fecal samples also containing fruit and those in which fruit was absent. Bar height indicates the proportion of samples containing a food type, and error bars show the 95% confidence intervals around those proportions. Asterisks indicate statistically significant differences in the probability of food types occurring in the diet when fruit is present or absent.

Generalized additive models revealed that the overall diet breadth of mandrills included approximately three distinct food types in an average fecal sample throughout the year, with slightly higher dietary diversity in the dry seasons (Figure 4A; Table SA9). An average diet breadth of three items and slightly more diversity in the dry seasons was remarkably consistent between years (Figure SA7), which is reflected in the narrow confidence interval around predictions for an average year in Figure 4A. In contrast to diet breadth, fruit breadth was observed to peak at roughly two genera of fruit in an average fecal sample in the two wet seasons, and to fall in the dry seasons; especially the long dry season, during which an average fecal sample was expected to contain only about one distinct fruit genus (Figure 4A; Table SA10). In addition, interannual variation in fruit breadth was substantial (Figure SA9), which is again reflected in the wide confidence interval around predictions for an average year in Figure 4B. Consumption of animal parts, crushed seeds, dicot leaves, and other fibers was negatively associated with the consumption of fruit. In contrast, there was no evidence that the consumption of monocot leaves and fungi was associated with fruit consumption (Table SA11, Figure 4B).

**Figure 5:**
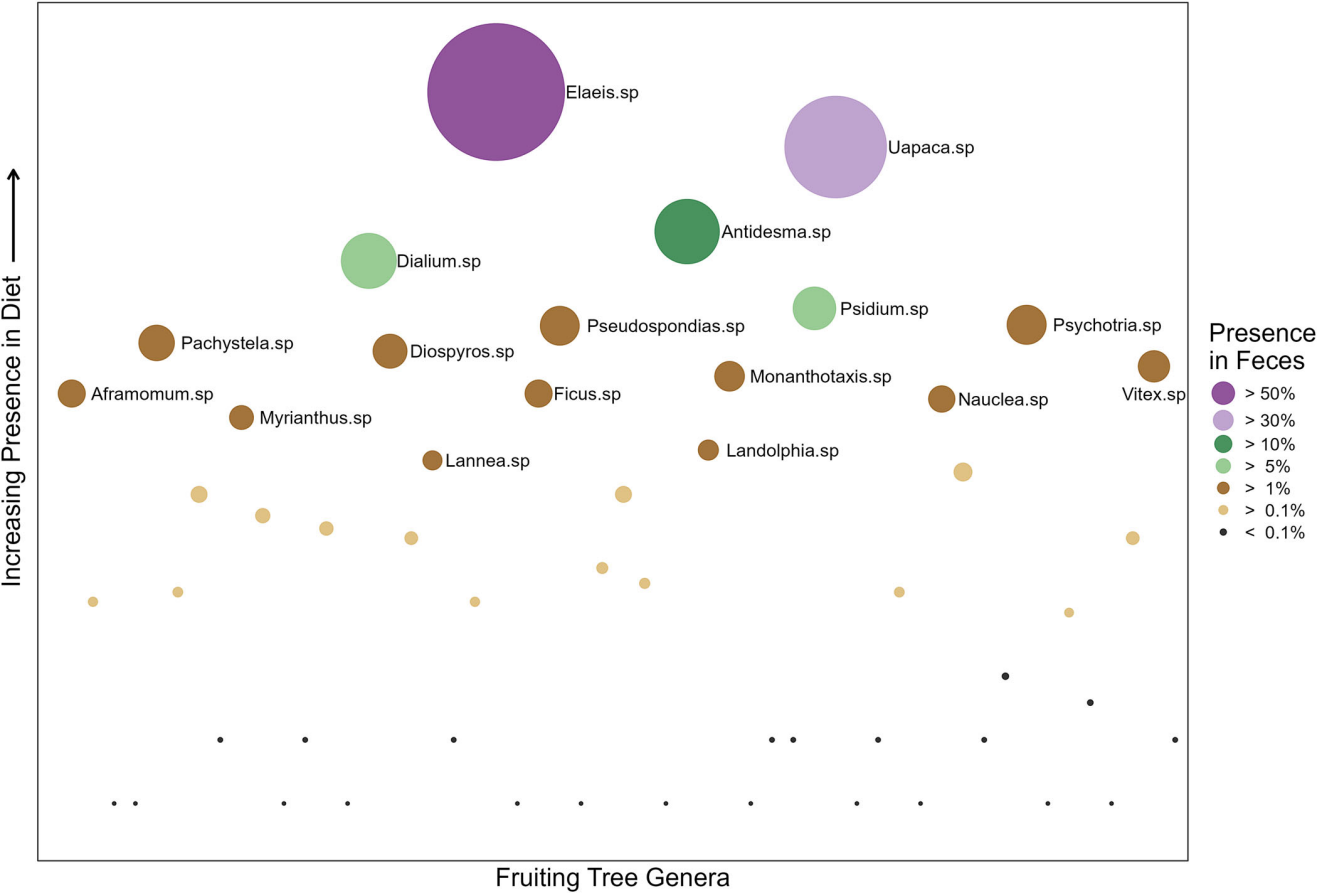
Fruit genera consumed by mandrills, ranked by presence in all 4024 fecal samples. Each point represents a single genus, dispersed arbitrarily on the *x*‐axis to avoid overlaps and ordered on the *y*‐axis by proportional occurrence in feces (log‐transformed).

**Figure 6:**
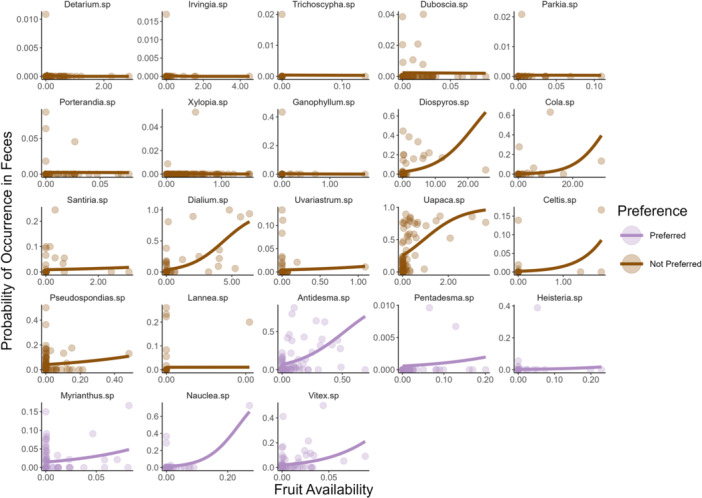
The relationship between fruit availability and consumption of fruiting genera by mandrills. Random slope predictions are shown over the observed availability range for each genus. Scales differ between facets to avoid compressing data for genera with lower availability. Intercepts of some species are > 0 because these fruits were consumed on the ground after the ripe fruit our phenology monitoring detects in canopies were no longer available. Effect sizes increase row‐wise from left to right, and column‐wise from top to bottom, with *Vitex* sp. exhibiting the largest effect size and *Detarium* sp. the smallest. Purple lines and dots indicate preferred genera that were consumed more than average as a function of availability, whereas brown lines and dots indicate non‐preferred genera that were consumed less than average as a function of availability.

We found fruit genera to appear in the diet at various frequencies (Figure 5). A generalized linear mixed model revealed a positive association between fruit availability and fruit consumption by mandrills across all genera tested (Table SA12). Likelihood ratio tests confirmed that goodness of fit was significantly greater for a model containing random slopes that allowed the relationship between availability and consumption to vary between fruiting genera than models containing only random intercepts for genera or only fixed effects (all *p* < 0.001). The random slopes for each genus are displayed in Figure 6 and indicate that, despite the overall positive association across all genera, there was substantial variation among taxa in the extent to which mandrill consumption covaries with availability. We found six genera to be consumed more frequently than average as a function of availability, suggesting the these genera are preferred by mandrills. Conversely, 17 genera were consumed less frequently than average as a function of availability, suggesting that these genera are not preferred by mandrills. Notably, the most favored genera were not always the most frequently consumed. For example, *Vitex* sp. were the most preferred genera, indicated by the steepest positive slope between availability and consumption, but were present in less than 5% of fecal samples. On the other hand, *Uapaca* sp. was present in over 30% of fecal samples, but only the tenth most preferred of the genera analyzed. In another comparison, *Detarium* sp. exhibited the weakest relationship between availability and consumption frequency and *Pentadesma* sp. the fifth strongest relationship, though both genera were present in less than 0.1% of fecal samples.

**Figure 7:**
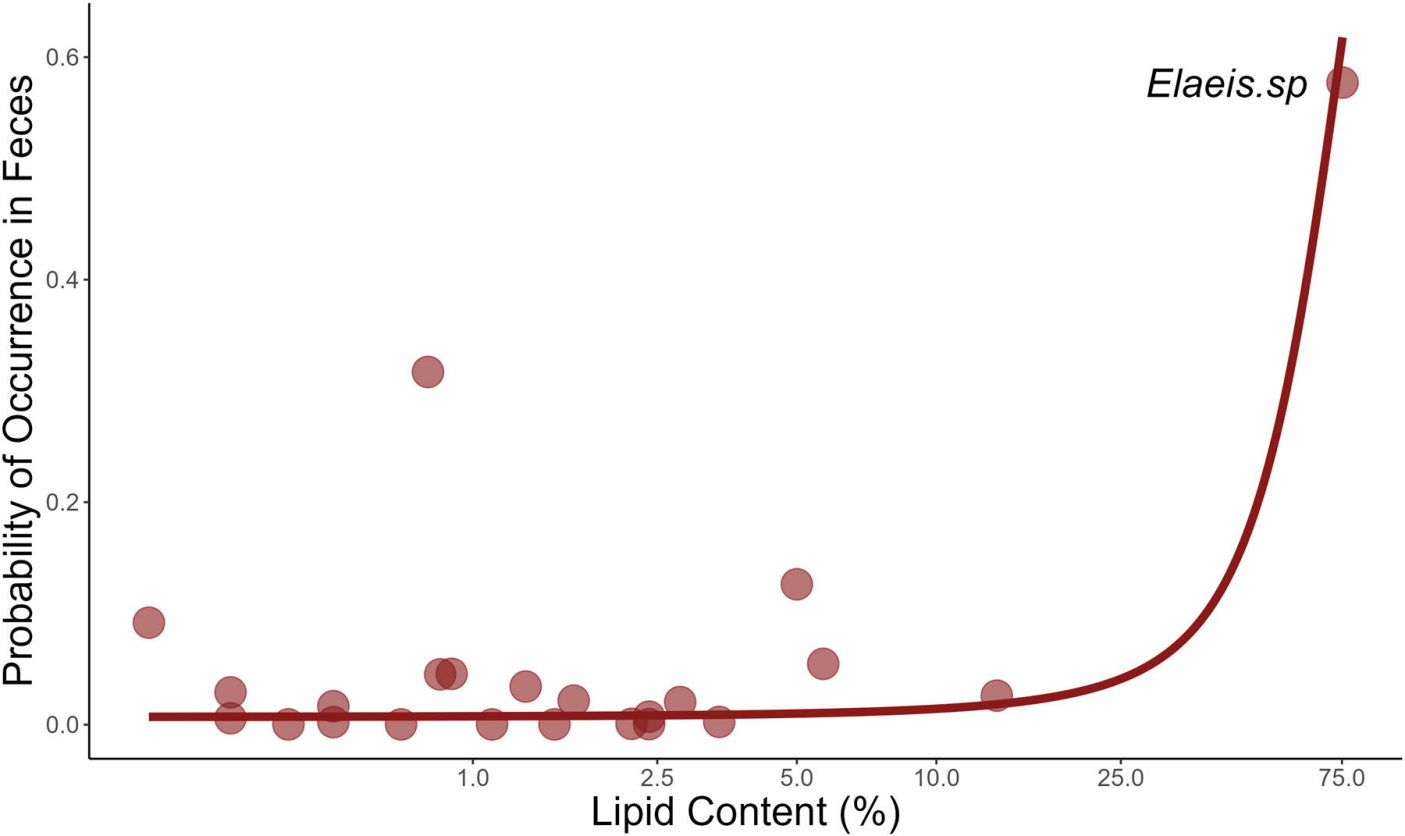
The relationship between maximum tree height and fruit consumption by mandrills. The *y*‐axis has been square root transformed and a jitter added to the data to better display overlapping points.

**Figure 8:**
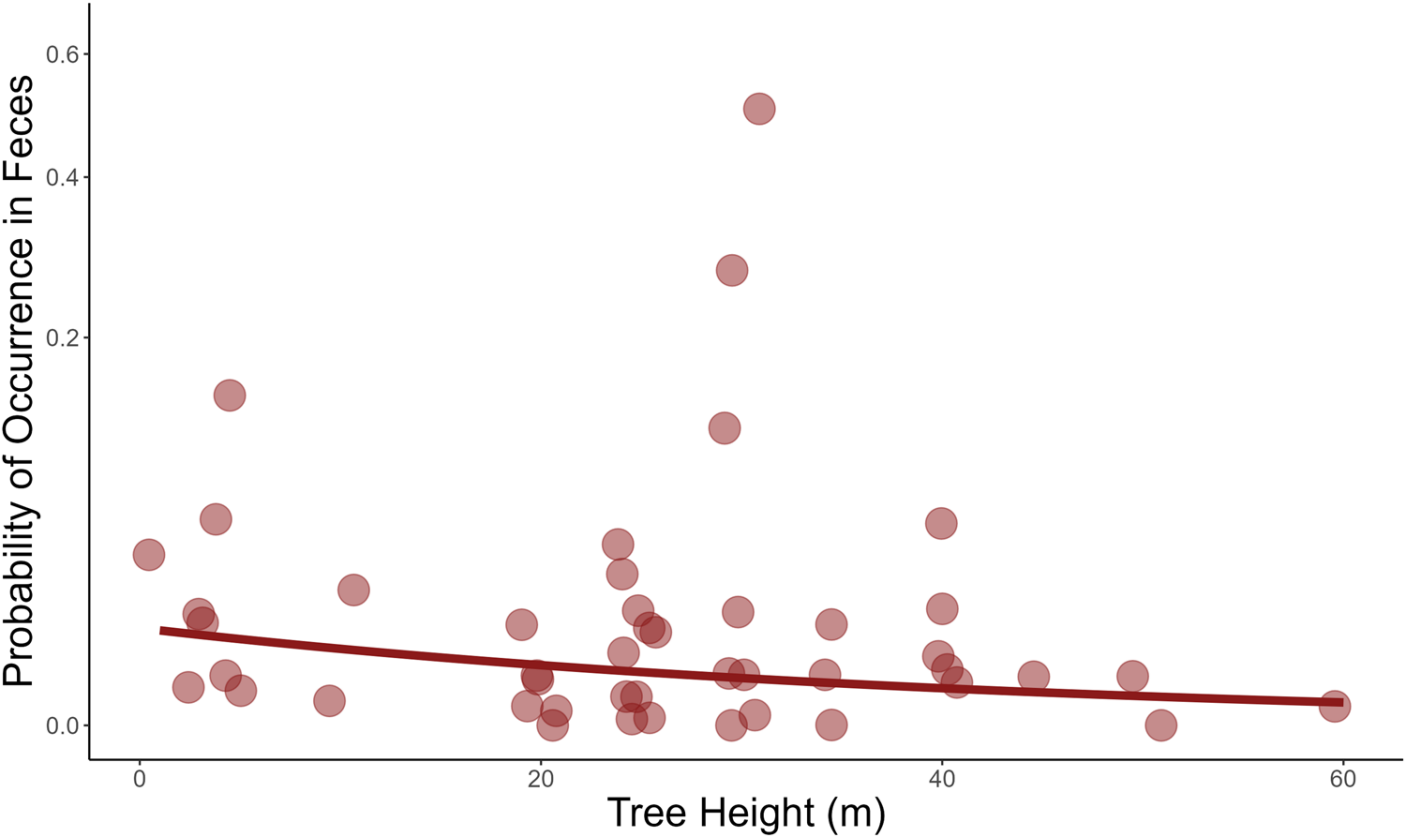
The relationship between fruit lipid content and consumption by mandrills. The *x*‐axis has been log^10^ transformed to spread clustered data at lower values. Removing *Elaeis* sp. (oil palm) from the data set resulted in no statistically significant relationship between lipid content and fruit consumption.

We used generalized linear mixed models to examine whether maximum tree height or fruit nutritional contents influenced mandrill fruit consumption. The association between tree height and fruit consumption was statistically significant and negative [−0.048, 95%CI: −0.086 to −0.011, *p* = 0.011] (Figure 7), while the association between fruit lipid contents and consumption was statistically significant and positive [0.072, 95%CI: 0.015–0.13, *p* = 0.014] (Figure 8). However, the association between lipid content and consumption appears to rely on a single high‐influence species of fruit: removing *Elaeis* sp. (oil palm) from the data set resulted in no statistically significant relationship between lipid content and fruit consumption. Associations between fruit consumption and contents of protein, carbohydrates, fiber, water, phenols, and tannins were not statistically significant (all *p* > 0.09).

## 4 Discussion

Using rare, long‐term data on mandrill feeding ecology and African tree phenology (Bush et al. 2017), we found that mandrills are highly frugivorous and that fruit consumption tracked availability. With generalized additive models, we also revealed that mandrill diet breadth increases when fruit availability falls due to fallback food consumption. Finally, we found variation between fruiting genera in the extent to which they are preferred by mandrills, at least insofar as consumption covaries with availability.

### 4.1 Mandrill Diet

Fruit was the most frequently present food type in mandrill fecal samples (Figure 2), and it exhibited two annual peaks corresponding to the west seasons when fruit availability is highest (Figure 3). Similarly, we found strong positive covariance between fruit availability and consumption (Table SA12). Together, these results indicate that fruit was the most preferred resource of our focal horde. We also found, however, that the seasonal timing of fruit consumption varied markedly between years in terms of both frequency (Figure SA1) and taxonomic breadth (Figure SA9). This interannual variation is likely due to differences between years in the timing and amount of fruit production in Lopé (Tutin et al. 1991; Bush, Whytock, et al. 2020), to which mandrills appear to respond by opportunistically consuming fruit when it is available.

In contrast to fruit, seasonal consumption of animal parts, dicot leaves, crushed seeds, and other fibers tended to peak when fruit consumption was relatively low (Figure 3). Again, there was substantial variation between years in the magnitude and seasonal timing of consumption of each of these alternative resources (Figure SA2:SA7). This interannual variation, coupled with negative covariance between consumption of these four food types and fruit consumption (Figure 4B), further suggests that mandrills feed opportunistically and that animal parts, dicot leaves, other fibers, and crushed seeds constitute important fallback foods. Furthermore, our finding that the overall diet breadth of mandrills consisted of three distinct food types throughout most seasons and across most years implies that generalist, opportunistic feeding is a stable behavior across time. In contrast to other fruit alternatives, we found no evidence that consumption of fungi or monocot leaves was negatively associated with fruit consumption (Figures 3 and 4B) and, therefore, no evidence that these food types function as important fallback food for mandrills.

Our results corroborate those of previous investigations indicating that mandrills are frugivorous omnivores whose diets exhibit seasonal responses to fruiting tree phenology (Rogers et al. 1996; Hongo et al. 2018). Given the 8‐year period during which our data were collected, a large number of dietary samples, and a robust measure of fruit availability (Bush et al. 2017; Cardoso et al. 2020), our results provide an atypically robust reflection of the diet of the focal horde. In turn, the general between‐study consistency in mandrill diets, across multiple locations (Jouventin 1975; Lahm 1986; White et al. 2010; Nsi Akoue et al. 2017; Hongo et al. 2018), supports their classification as highly generalist frugivore‐omnivores. Further study using advanced techniques, such as stable isotope analysis (Crowley 2012) or metagenomics (Srivathsan et al. 2016) may prove useful for identifying the full taxonomic diversity of mandrill diets, or for describing the impacts of extreme dimorphism and group size on mandrill feeding ecology. For example, Oelze et al. (2020) identified age‐sex class differences in feeding and nutritional stress using stable isotopes. Furthermore, species such as *Pentaclethra macrophyla* are known to be eaten by the focal horde, but the large seeds could not be identified in fecal samples because they are crushed during mastication.

### 4.2 Mandrill Nutritional Strategy

The feeding strategies employed by primates are thought to have evolved to allow sufficient micro‐ and macro‐nutrients to be obtained from their habitat (Felton, Felton, Lindenmayer, et al. 2009). For example, spider monkeys (*Ateles chamek*) and chimpanzees (*Pan troglodytes*) appear to prioritize protein intake, balancing their consumption of carbohydrates and lipids accordingly (Felton, Felton, Raubenheimer, et al. 2009; Uwimbabazi et al. 2021). Conversely, mountain gorillas (*Gorilla beringei*) living in a protein‐rich habitat target foods, allowing energy intake to be maximized through sugars (Ganas et al. 2008; Rothman et al. 2008). To our knowledge, no formal analyses of mandrill nutritional ecology are available, so we also explored whether the preferences of our focal horde were related to fruit nutritional contents.

Our data indicated that some fruits were consumed more than others as a function of availability (Figure 6), suggesting preference by the focal horde for certain fruit genera. Notably, we found some fruiting genera to be consumed at higher rates than average, suggesting that these are relatively preferred. Conversely, other genera were consumed less frequently than average, suggesting that these fruits are not preferred by mandrills even if, in some cases, they are among the most commonly consumed (e.g., *Uapaca* sp., Figure 6). These preferences are likely to be driven by the nutritional contents of particular fruits (Felton, Felton, Lindenmayer, et al. 2009; Felton, Felton, Raubenheimer, et al. 2009). However, in our analyses of the relationship between fruit nutritional contents and consumption, we found a statistically significant association only between lipid content and consumption frequency. The significant association we found, however, was driven largely by the consumption of oil palm fruits, which were by far the most frequently consumed resource (Figure 5) and contain 75% lipids. Such high consumption frequencies suggest that oil palm fruits are a staple resource for our focal mandrill horde, as noted for other primates in our study site (Tutin et al. 1997) and for West African chimpanzee populations (Garriga et al. 2019; Bryson‐Morrison et al. 2020). The importance of oil palm fruit to primates in our study site is consistent with the designation of palm fruits generally as a keystone resource that may have played a role in the evolution color vision in African primates (Onstein et al. 2020).

Unfortunately, we did not have abundance data for palm fruits, but palms are known to fruit year‐round in the study site (White 2007). Oil palms may, therefore, comprise an energy source for our focal horde that is consistently available throughout the year. If energy from palm fruits is targeted to a greater degree, the year‐round availability of lipid‐rich fruits may explain why we found no meaningful association between carbohydrate content (an alternate source of energy) and fruit consumption. More specifically, an abundance of available lipid‐rich palm fruits may have reduced the need for mandrills to pursue caloric intake in the form of carbohydrates. We also did not find an association between fruit protein content and consumption frequency. A low influence of protein content on mandrill fruit selection could arise because fruits are typically low in protein compared to other food items that primates feed on (Rothman et al. 2014). Thus, individuals in the focal horde may not have selected fruits due to protein content because they gain more substantial amounts of protein from other food sources. Notably, we found peaks in animal part consumption during the two wet seasons, when fruit consumption is also frequent (Figure 3) and animal prey often contributes a substantial amount of protein to primate macronutrient intake (O'Malley and Power 2014; Bryer et al. 2015).

We did not find statistically significant relationships between fruit consumption and contents of fiber, water, tannins, or phenols. We, therefore, did not find evidence of factors outside of macronutrients, such as plant secondary compounds, affecting fruit selection by mandrills. The consumption of fruit containing defensive compounds may form a part of mandrills' extremely generalist feeding strategy if they are less selective than other primates in terms of fruit quality. Alternatively, nutritional data on a greater range of fruit species may be needed to further examine the effect of secondary compounds. We only had nutritional contents data for a subset of the fruit genera consumed by our focal horde, and it would therefore be useful to increase the taxonomic coverage of our data set, to carry out a more complete analysis of the nutritional ecology of the focal horde. It would also be interesting to compare the nutritional ecology of mandrills in different locations or habitat types to examine the effects of the presence or absence of particular resources. For example, oil palms are abundant in Lopé but not in other locations (e.g., Lékédi, Nsi Akoue et al. 2017; Moukalaba‐Doudou, Hongo et al. 2018). It would, therefore, be informative to examine how the presence of lipid‐rich oil palm fruits impacts food selection and other aspects of mandrill behavior, including between site variation in observed group size. Nutritional analyses may also reveal the potential fitness consequences of obtaining preferred versus fallback foods and give insights into morphological trait evolution and the resilience of mandrills to environmental change.

### 4.3 Feeding Competition

The numbers of mandrills present in a horde, the highest of any non‐human primate (Abernethy et al. 2002) mean that the food demands of a single group are extraordinarily high. The biomass of the mandrill horde is around 4852 kg (White et al. 2010), roughly equivalent to the average group biomass for forest elephants at the site (4876 kg; White 1994) and close to 12 times the group biomass for the largest frugivorous primate, the lowland gorilla (414 kg; White 1994). These high food demands, as well as rapid rates of patch depletion, are the most likely explanation for mandrills also exploiting the largest home ranges observed in wild primates (White et al. 2010). It is also probably the case that the dietary generalism documented here and elsewhere (Hongo et al. 2018) is, in part, an adaptation to extreme group sizes. We have documented that the diversity of food types eaten by mandrills increases when fruit availability is low. This ability to switch resources in response to a lack of fruit likely carries over to when individual animals are unable to access fruit in feeding patches. Thus, dietary generalism may allow individuals to continue to intake resources even when they lose out during indirect competition within a mandrill horde. Mandrills are also the most sexually dimorphic primate (Setchell 2016), and sex differences in diet have been observed in other study sites, such as males consuming more hard foods than females (Nsi Akoue et al. 2017; Percher et al. 2017). Therefore, it could also be the case that mandrill dimorphism facilitates some degree of niche differentiation between sexes that helps to alleviate feeding competition in large social groups.

Mandrills must also cope with interspecific feeding competition. In Lopé, mandrills coexist with several other frugivores (11 other monkey species, chimpanzees, gorillas, red river hogs, and forest elephants), and our focal horde's dietary niche may be influenced by feeding competition and resource partitioning among species. Segregating habitats by height is one way in which coexisting frugivores may attempt to alleviate feeding competition (Sushma and Singh 2006). Mandrills are often considered semi‐terrestrial primates, as opposed to purely arboreal, because they forage mostly, but not exclusively, at ground level (Hoshino 1985). However, the negative association we found between maximum tree height and fruit consumption was relatively weak (Figure 7), and so does not suggest that tree height greatly limits mandrill access to certain fruits or that arboreal habitats are avoided. Notably, oil palm and *Uapaca* sp. are medium‐sized trees (within our sample) and their fruits were more frequently consumed than any others. Furthermore, because we found only a modest effect of maximum height, and most trees are necessarily shorter than the species maximum height, it is very unlikely that mandrills are limited in terms of foraging height throughout most of their habitat.

Alongside arboreal foraging, mandrills also spend substantial amounts of time feeding at ground level within leaf litter (Rogers et al. 1996). Consumption of fallen fruit and seeds at ground level most likely explains why we often observed consumption of some fruit species when our phenology data indicated zero availability of these species in the canopy (Figure 6). Our focal horde also exploited a 118 km^2^ (46 km^2^ of forest) home range during the same period as feces were collected (White et al. 2010). This estimate is much larger than those for sympatric frugivores at other sites in Central Africa, such as forest elephants at 75 km^2^ (Blake et al. 2008), gorillas at 38 km^2^ (Sanz 2004), and chimpanzees at 18 km^2^ (Cipolletta 2004). A prolonged period of fruit scarcity in 2003 also appeared to result in increased group‐fissioning by the focal horde, reducing the size of subgroups, with subgroups breaking away from the horde to forage in other areas of the home range (White 2007). Mandrills were also found to travel further per day as fruit availability decreased (White 2007). High mobility and social plasticity, alongside the dietary generalism documented here and by others (Rogers et al. 1996; Nsi Akoue et al. 2017; Hongo et al. 2018), may all play a role in allowing mandrills to coexist with many competing species. Examining the exact nature of feeding competition (and facilitation) between sympatric frugivores in Gabon would be another interesting avenue for future study.

### 4.4 Mandrill Conservation

Evaluating the resilience of mandrills to environmental change is necessitated by their classification as Vulnerable on the IUCN Red List, with habitat degradation from climate change one of the threats identified (Abernethy and Maisels 2019). The high social and dietary plasticity of mandrills may be a response to the extreme variation in resource availability in West Central Africa over the past millennia (Maley 1996; White 2001). Dietary flexibility, in the form of resource switching, could therefore confer some level of resilience to climate change‐induced falls in fruit production (Korstjens and Hillyer 2016). However, mandrill hordes are so large, with an approximate biomass of 4.1 tons (derived from Abernethy et al. 2002), that availability of alternative foods may be limited. At our study site, climate change over the past three decades has resulted in a 1°C rise in temperature, alongside a 300 mm fall in annual rainfall and a longer dry season (Bush, Jeffery, et al. 2020). Consequently, fruit availability has fallen dramatically at Lopé in the last 30 years (Bush, Whytock, et al. 2020).

For comparison, forest elephants, the largest mammal found at Lopé and also a frugivore, have a comparable mean group biomass of around 4.8 tons (White 1994). A comparison of recent and historical elephant fecal samples from Lopé indicated that elephants are consuming less fruit and much greater quantities of low‐quality, fibrous plant tissues in 2022 than they did in 1990 (Tejler et al. unpublished data). This shift in diet, likely a response to lower fruit availability, has been linked to increased seasonal emaciation of elephants documented at Lopé (Bush, Whytock, et al. 2020), suggesting that elephants have been unable to maintain the nutritional quality of their diet faced with current levels of tree productivity. As our fecal data were gathered between 1996 and 2004, they could provide a useful baseline for comparison to newer data, to examine whether the Lopé mandrills' diet has changed in the intervening 20 years. Contemporary data could highlight whether mandrills are consuming the same fruits as before or whether they are having to consume fruit that we found to be less preferred. Additionally, repeating our analysis could indicate whether temporal consumption of the fruit has remained consistent or if fallback foods are forming a greater part of present‐day mandrill diets.

## Author Contributions

**Joshua Bauld:** conceptualization (equal), data curation (equal), formal analysis (equal), investigation (equal), methodology (equal), software (equal), validation (equal), visualization (equal), writing–original draft (equal), writing–review and editing (equal). **David Lehmann:** conceptualization (equal), formal analysis (equal), investigation (equal), methodology (equal), software (equal), supervision (equal), visualization (equal), writing– original draft (equal), writing–review and editing (equal). **Luc F. Bussière:** conceptualization (equal), data curation (equal), formal analysis (equal), investigation (equal), methodology (equal), software (equal), supervision (equal), validation (equal), visualization (equal), writing–original draft (equal), writing–review and editing (equal). **Emma R. Bush:** data curation (equal), formal analysis (equal), methodology (equal), writing–review and editing (equal). **Edmond Dimoto:** conceptualization (equal), data curation (equal), investigation (equal), methodology (equal), writing–review and editing (equal). **Jean‐Thoussaint Dikangadissi:** conceptualization (equal), data curation (equal), investigation (equal), methodology (equal), writing–review and editing (equal). **Tharcisse Ukizintambara:** conceptualization (equal), data curation (equal), investigation (equal), methodology (equal), writing–review and editing (equal). **Elizabeth C. White:** conceptualization (equal), data curation (equal), funding acquisition (equal), investigation (equal), methodology (equal), project administration (equal), writing–review and editing (equal). **Jason Newton:** conceptualization (equal), formal analysis (equal), investigation (equal), methodology (equal), supervision (equal), visualization (equal), writing–original draft (equal), writing–review and editing (equal). **Isabel L. Jones:** formal analysis (equal), investigation (equal), methodology (equal), supervision (equal), visualization (equal), writing–original draft (equal), writing–review and editing (equal). **Lee J. T. White:** conceptualization (equal), data curation (equal), funding acquisition (equal), investigation (equal), methodology (equal), project administration (equal), writing–review and editing (equal). **Ruth Musgrave:** conceptualization (equal), data curation (equal), investigation (equal), methodology (equal), writing–review and editing (equal). **Kate A. Abernethy:** conceptualization (equal), data curation (equal), formal analysis (equal), funding acquisition (equal), investigation (equal), methodology (equal), project administration (equal), supervision (equal), visualization (equal), writing–original draft (equal), writing–review and editing (equal).

## Ethics Statement

The collection of mandrill fecal samples was permitted by the CIRMF Scientific Council (Gabon) and the Direction of Wildlife (Gabon) and was consistent with the American Society of Primatologists Principles for the Ethical Treatment of Non‐Human Primates.

## Supporting information

Supporting information.

## Data Availability

The mandrill dietary data that support the findings of this study are available from the corresponding author upon reasonable request. The Lopé Tree Phenology Data set is available at: http://hdl.handle.net/11667/152.
