# Supplementary material for: Rare Long‐Term Data Reveal the Seasonal Dietary Plasticity of Mandrills (Mandrillus sphinx) in Response to Fruiting Tree Phenology
Source: Am J Primatol. 2025 Mar 17;87(3):e70012. doi: 10.1002/ajp.70012 (PMC11913773; doi:10.1002/ajp.70012)
Supplement: Supplementary file 1 — Supporting information. [file AJP-87-e70012-s001.docx]

**Appendix**
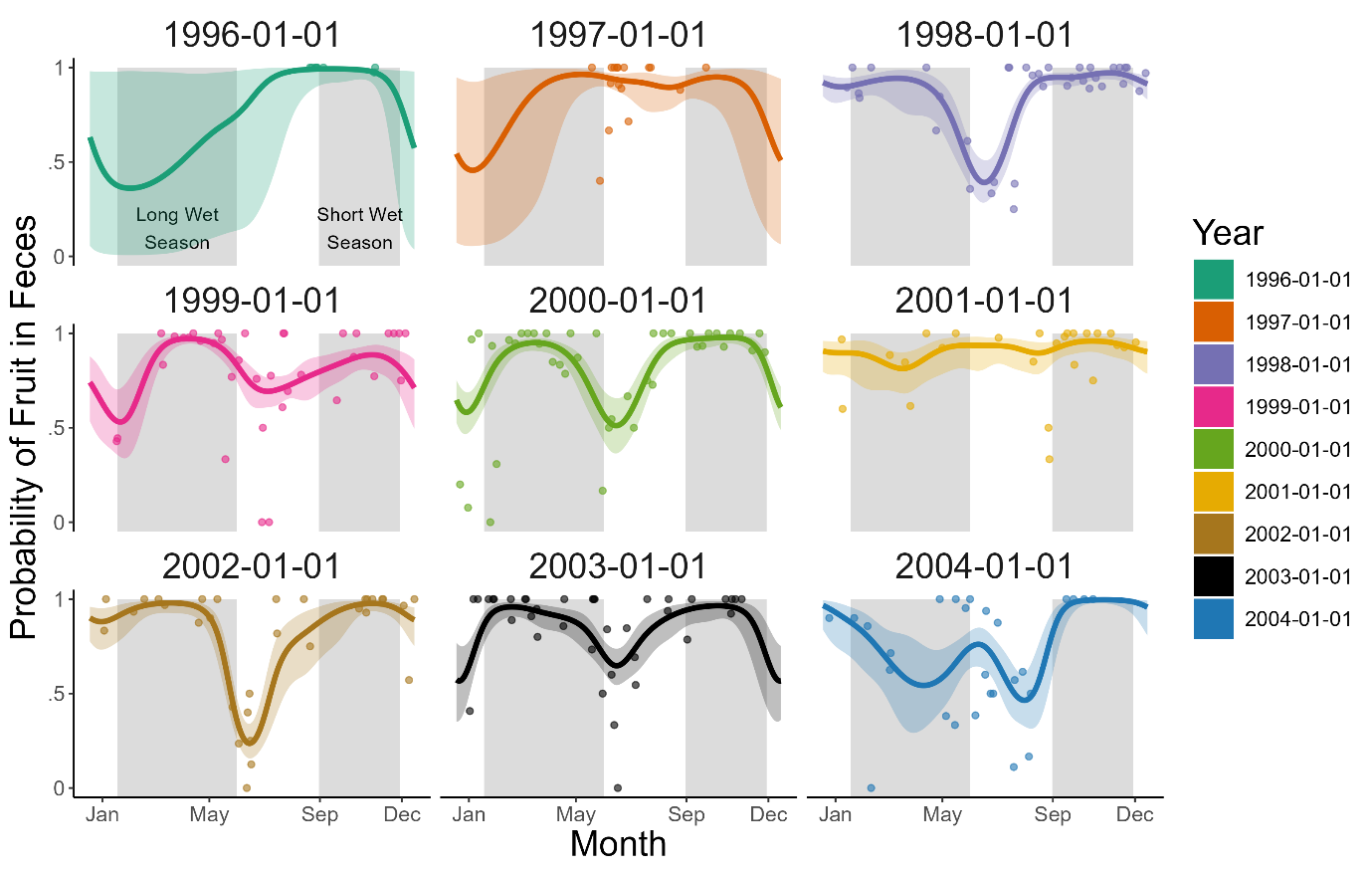
**Figure A1.** Inter-annual variance in the relationship between Day of Year and the probability of fruit presence in mandrill fecal samples. Lines illustrate the random smooths for individual years, band display the 95% confidence intervals around the predictions and points show the observed fruit presence in fecal samples collected on a given day of the year.

**
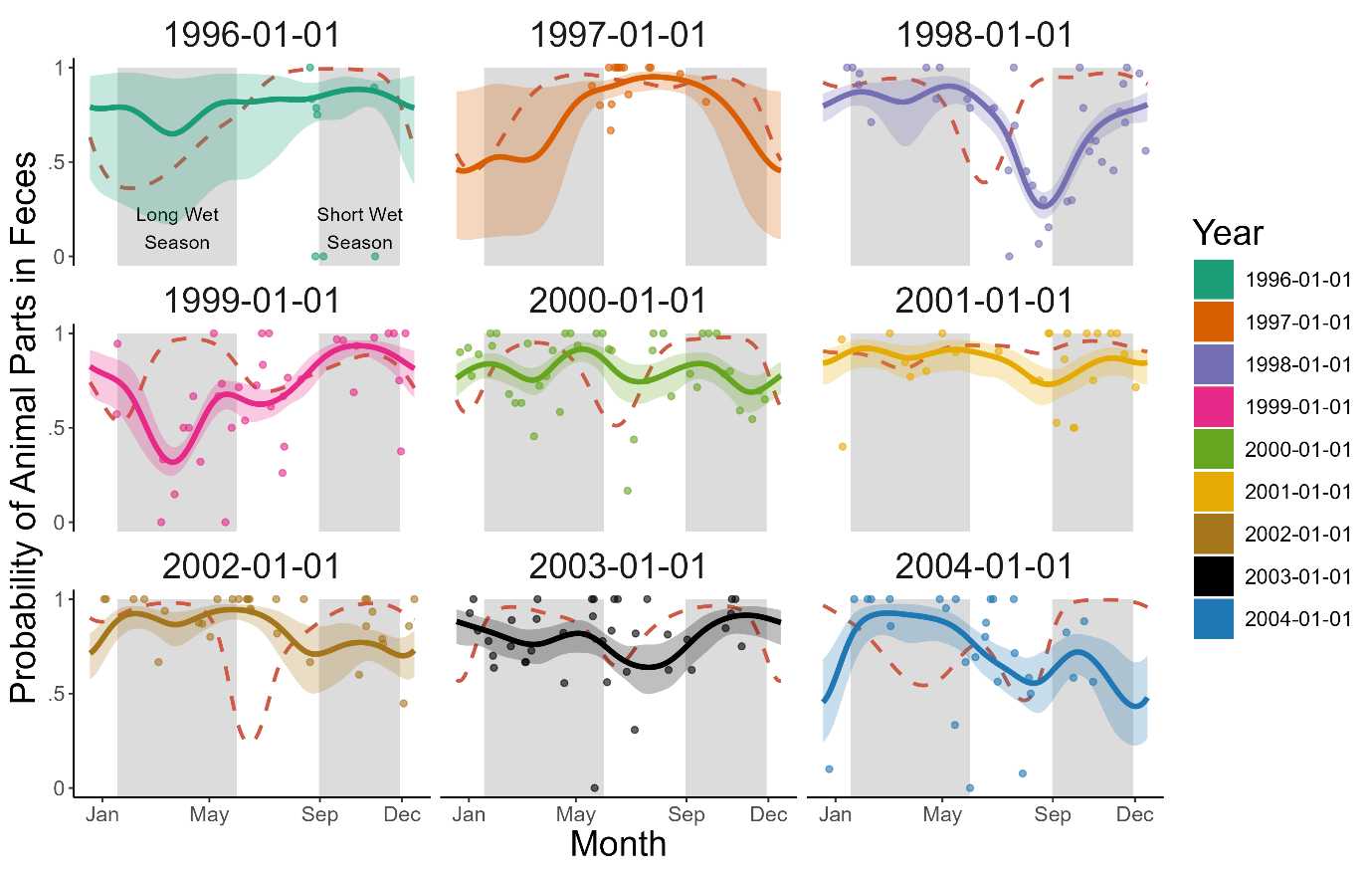
**

**Figure A2.** Inter-annual variance in the relationship between Day of Year and the probability of animal parts presence in mandrill fecal samples. Lines illustrate the random smooths for individual years, band display the 95% confidence intervals around the predictions and points show the observed animal part presence in fecal samples collected on a given day of the year. The dashed orange line displays inter-annual variation in fruit consumption for comparison.

**
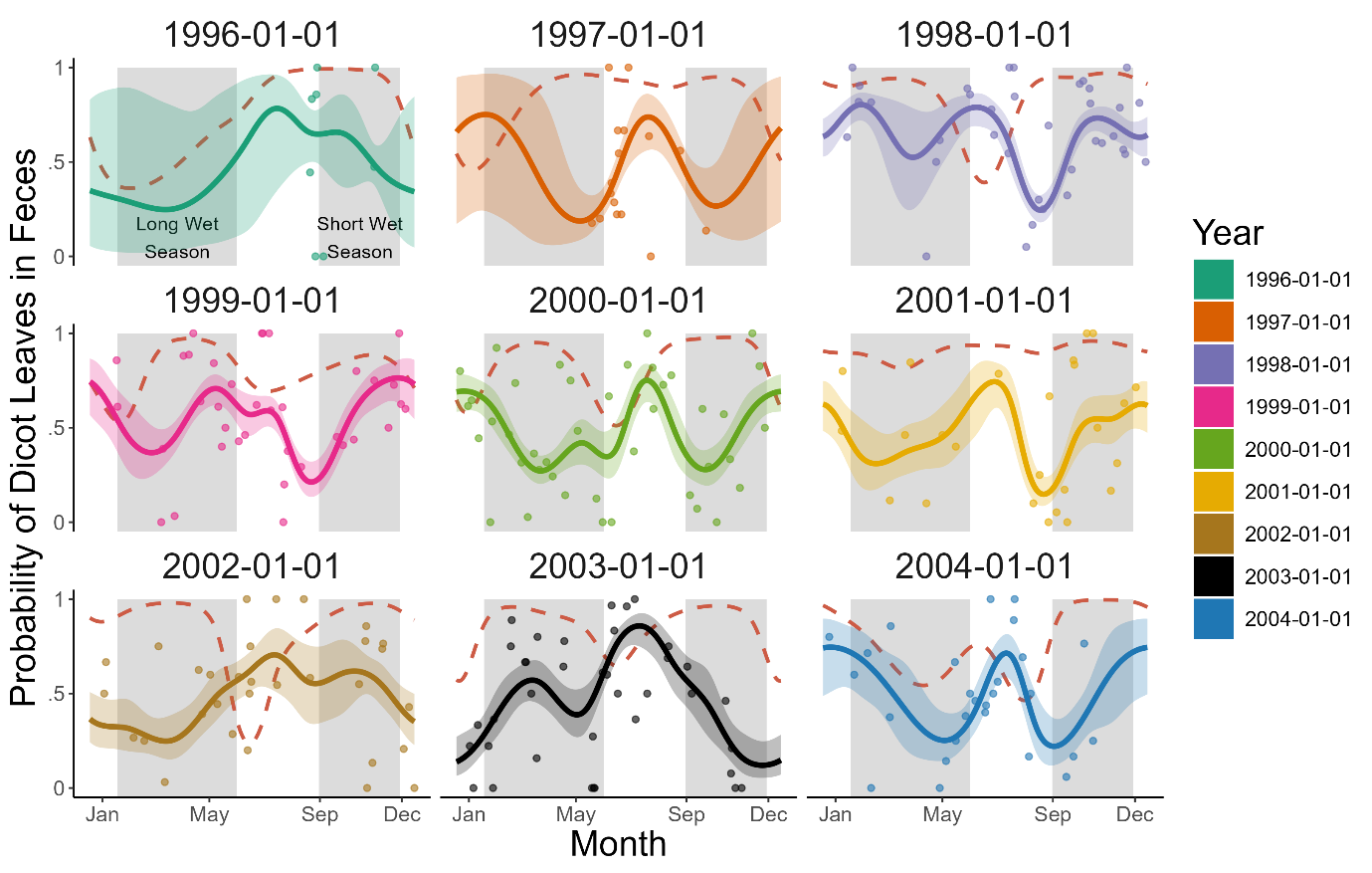
**

**Figure A3.** Inter-annual variance in the relationship between Day of Year and the probability of dicot leaf presence in mandrill fecal samples. Lines illustrate the random smooths for individual years, band display the 95% confidence intervals around the predictions and points show the observed dicot leaf presence in fecal samples collected on a given day of the year. The dashed orange line displays inter-annual variation in fruit consumption for comparison.

**
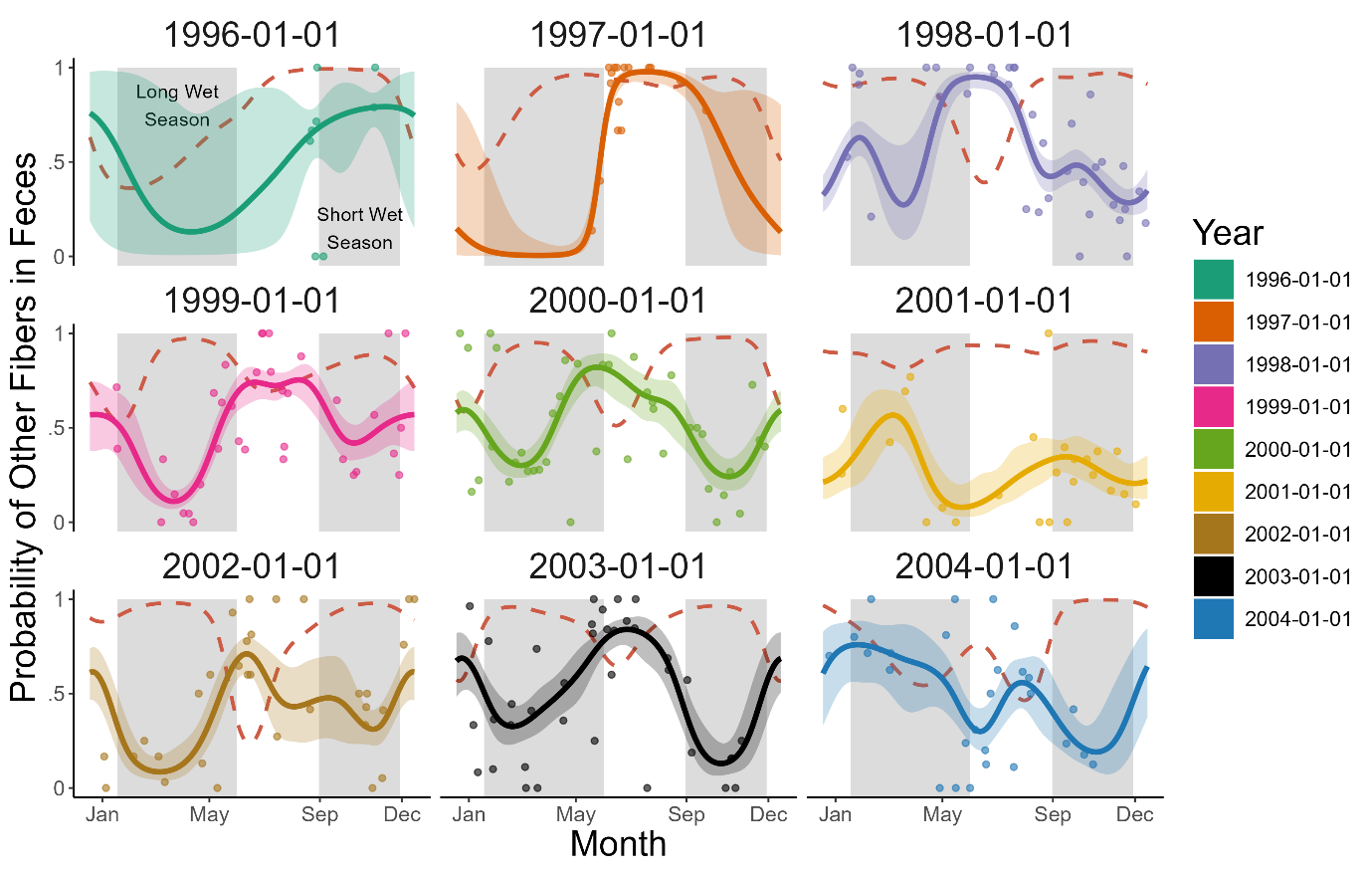
**

**Figure A4.** Inter-annual variance in the relationship between Day of Year and the probability of other fiber presence in mandrill fecal samples. Lines illustrate the random smooths for individual years, band display the 95% confidence intervals around the predictions and points show the observed other fiber presence in fecal samples collected on a given day of the year. The dashed orange line displays inter-annual variation in fruit consumption for comparison.

**
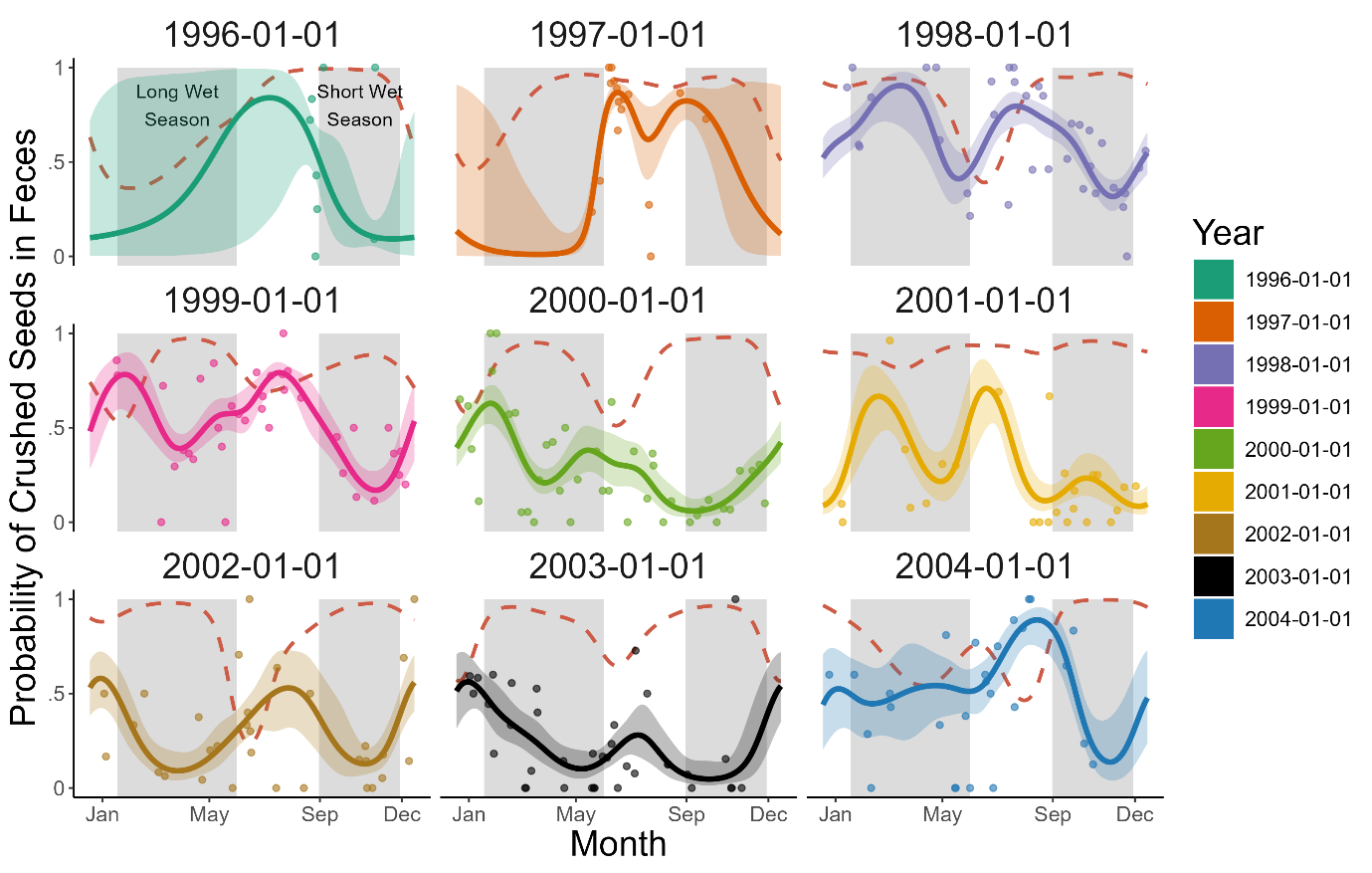
**

**Figure A5.** Inter-annual variance in the relationship between Day of Year and the probability of crushed seed presence in mandrill fecal samples. Lines illustrate the random smooths for individual years, band display the 95% confidence intervals around the predictions and points show the observed crushed seed presence in fecal samples collected on a given day of the year. The dashed orange line displays inter-annual variation in fruit consumption for comparison.

**
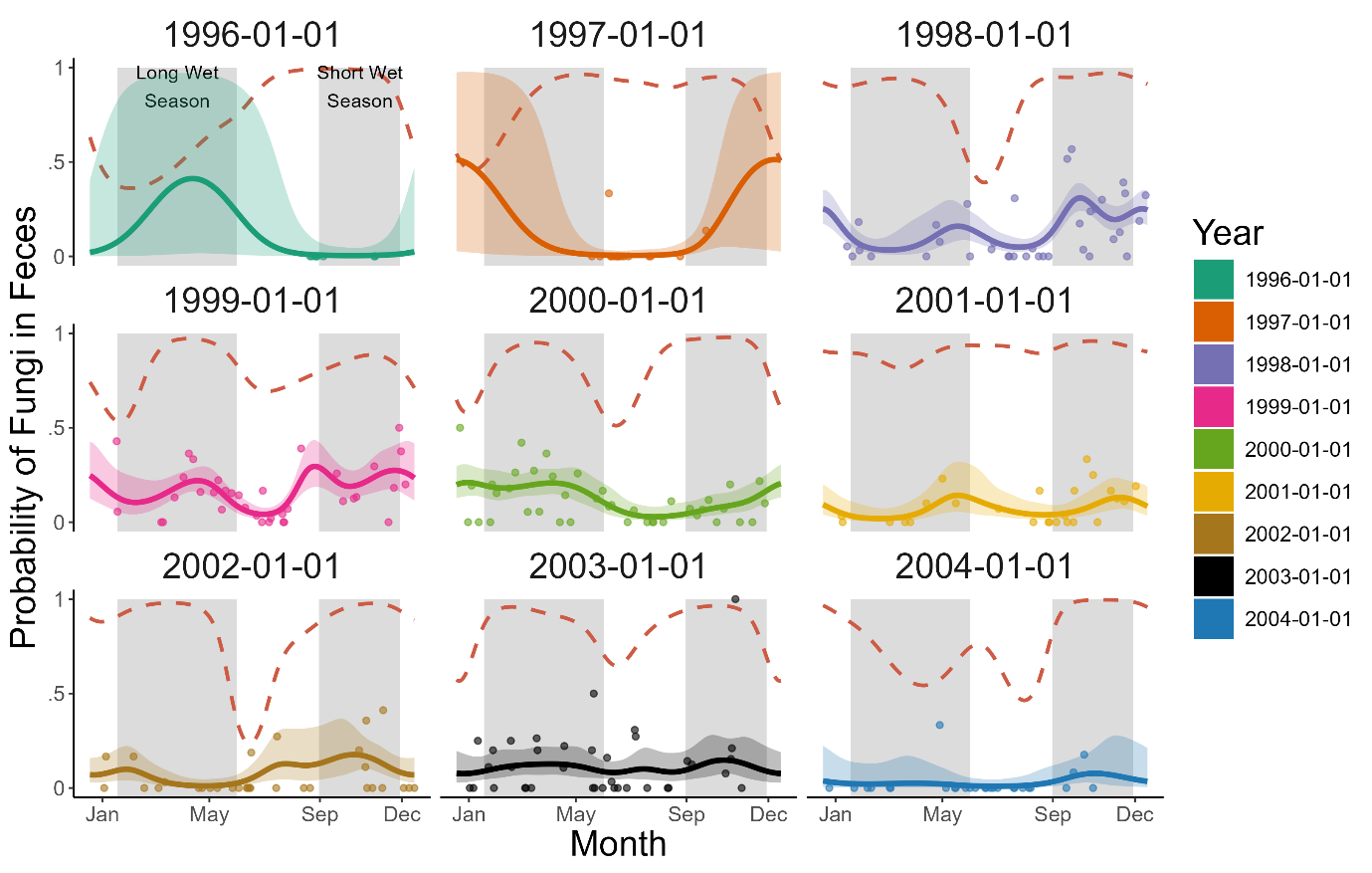
**

**Figure A6.** Inter-annual variance in the relationship between Day of Year and the probability of fungi presence in mandrill fecal samples. Lines illustrate the random smooths for individual years, band display the 95% confidence intervals around the predictions and points show the observed fungi presence in fecal samples collected on a given day of the year. The dashed orange line displays inter-annual variation in fruit consumption for comparison.

**
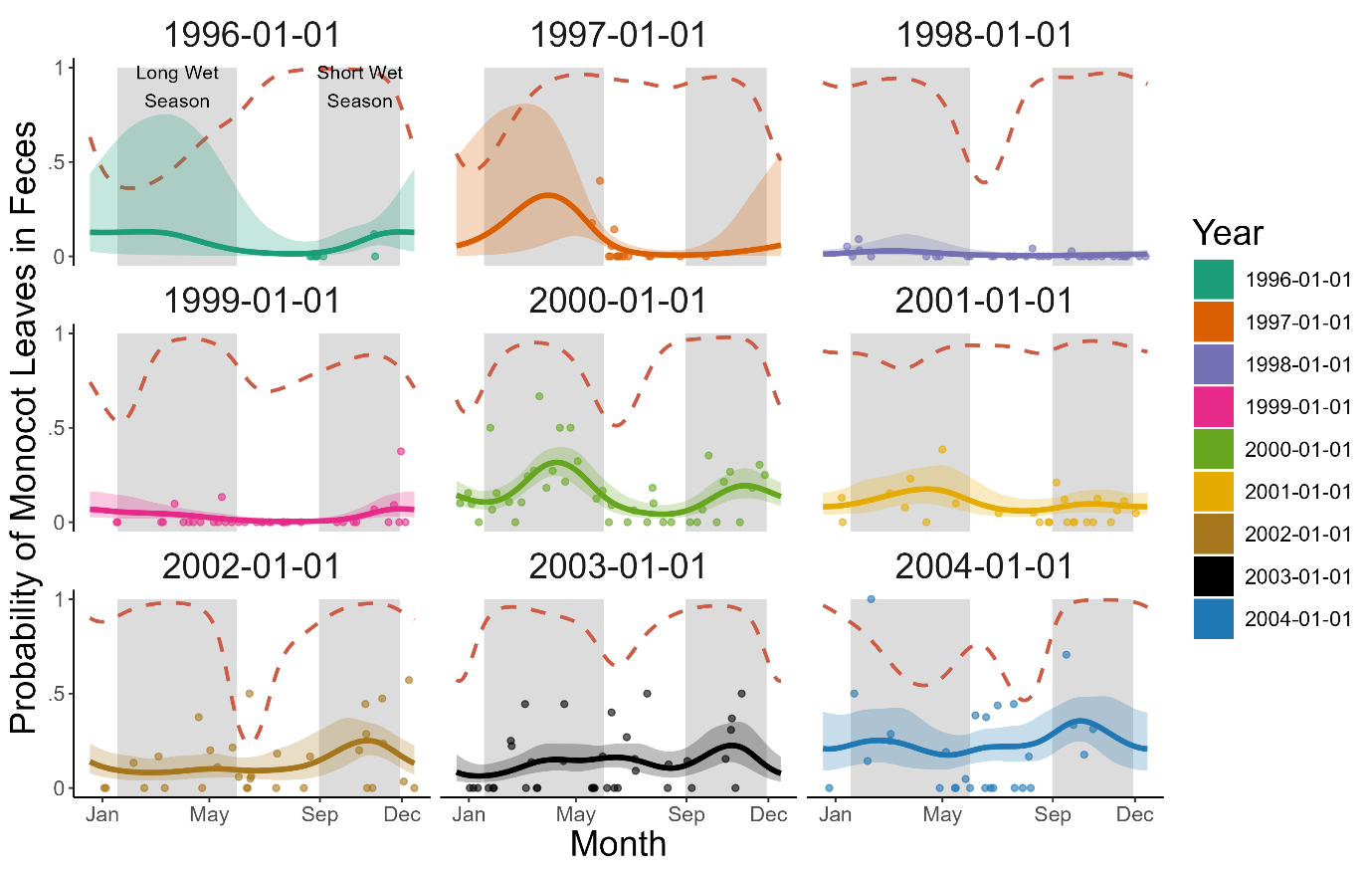
**

**Figure A7.** Inter-annual variance in the relationship between Day of Year and the probability of monocot leaf presence in mandrill fecal samples. Lines illustrate the random smooths for individual years, band display the 95% confidence intervals around the predictions and points show the observed monocot leaf presence in fecal samples collected on a given day of the year. The dashed orange line displays inter-annual variation in fruit consumption for comparison.

**
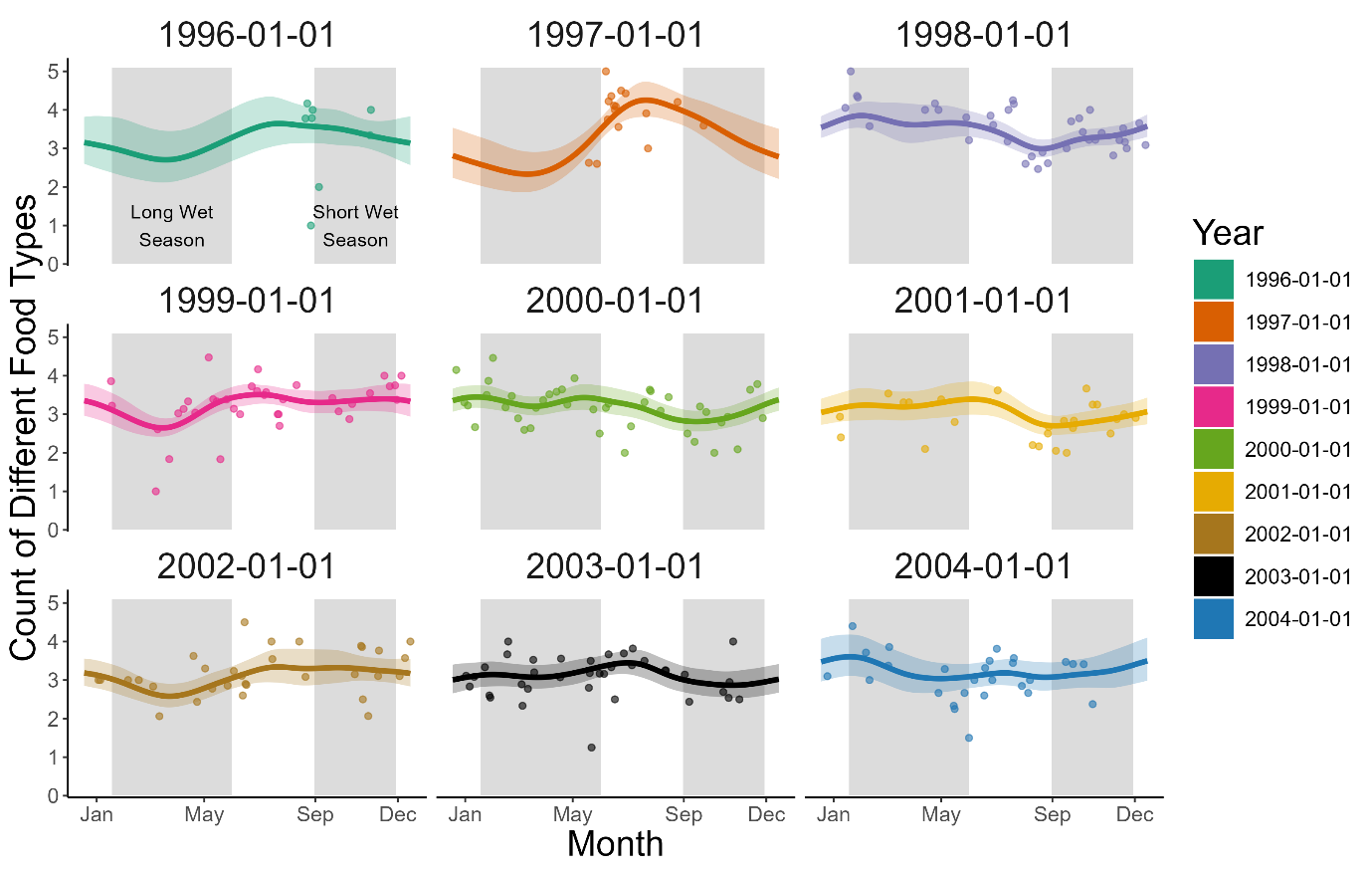
**

**Figure A8.** Inter-annual variance in the relationship between Day of Year and diet breadth, i.e. the count of different food types present in mandrill fecal samples. Lines illustrate the random smooths for individual years, band display the 95% confidence intervals around the predictions and points show the mean count of different food types in fecal samples collected on a given day of the year.

**
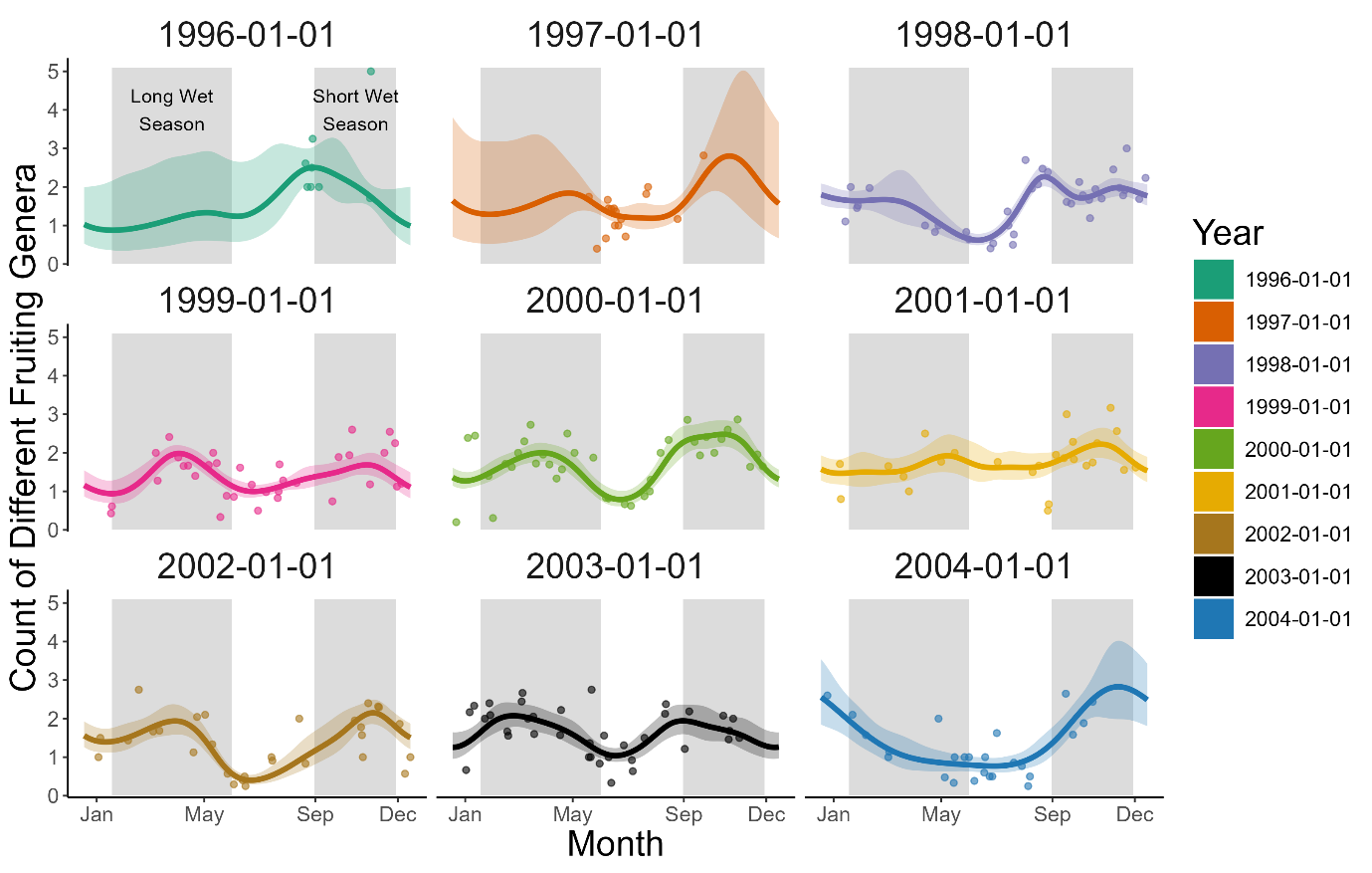
**

**Figure A9.** Inter-annual variance in the relationship between Day of Year and fruit breadth, i.e. the count of different fruiting genera present in mandrill fecal samples. Lines illustrate the random smooths for individual years, band display the 95% confidence intervals around the predictions and points show the mean count of fruit genera in fecal samples collected on a given day of the year.

**Table A1.** All plant taxa identified in mandrill feces. The lowest taxonomic level identified in each family is highlighted in bold. Where a genus was only represented by a single species in Lope National Park, this species was taken as present in the diet. Where no members of a genus or family were identified to species level, the genus or family was taken as present in the diet. This resulted in a minimum dietary diversity of 67 identified plant taxa and a maximum dietary diversity of 119 plant taxa, if all species of each family found in mandrill feces were assumed to be eaten. In addition to these 119 taxa, species known only from observational data to be consumed by the group are highlighted in red, meaning the total diversity of taxa consumed by mandrills is higher than we found through fecal analysis.

| Family | Genus | Species |
| --- | --- | --- |
| Zingiberaceae | *Aframomum* | ***sericeum,*** *longipetiolatum, leptolepsis* |
| Annonaceae | *Annonidium* | ***floribundum*** |
| Euphorbiaceae | *Antidesma* | ***venosum, vogelianum*** |
| Lauraceae | *Belschmeidia* | ***fulva*** |
| Ochnaceae | *Campylospermum* | *elongatum* |
| Zingiberaceae | *Costas* | *afer* |
| Ulmaceae | *Celtis* | ***tessmannii*** |
| Vitaceae | *Cissus* | ***dinklagei*** |
| Rutaceae | *Citrus* | ***limonum*** |
| Sterculiaceae | *Cola* | ***lizae*** |
| Olacaceae | *Coula* | ***edulis*** |
| Euphorbiaceae | *Croton* | ***mubango*** |
| Leguminosae | *Cryptocephalum* | ***staudtii*** |
| Leguminosae | *Detarium* | ***macrocarpum*** |
| Leguminosae | *Dialium* | ***lopense, pachyphyllum,*** *guineense, dinklagei, soyauxii* |
| Ebenaceae | *Diospyros* | ***dendo, manni, polystemon, zenkeri*** |
| Euphorbiaceae | *Discoglypremna* | ***caloneura*** |
| Sapotaceae | *Donella* | ***ogowensis*** |
| Tiliaceae | *Duboscia* | ***macrocarpa*** |
| Arececeae | *Elaeis* | ***guineensis*** |
| Annonaceae | *Enantia* | ***chlorantha*** |
| Moraceae | ***Ficus*** | *barteria, carica, cyathistipuloides, elasticoides, kimuenzensis, mucuso, ovata, polita, recurvata, subsagittifolia, sur, thonningii, variifolia* |
| Sapotaceae | *Gambeya* | ***subnuda*** |
| Sapindaceae | *Ganophyllum* | ***giganteum*** |
| Clusiaceae | *Garcinia* | ***gnetoides, ovalifolia*** |
| Olacaceae | *Heisteria* | ***parvifolia*** |
| Acanthaceae | *Hypoestes* | *verticullaris* |
| Irvingiaceae | *Irvingia* | ***gabonensis*** |
| Irvingiaceae | Klainedoxa | *gabonensis* |
| Apocynaceae | *Landolphia* | ***incerta, jumellei, manni*** |
| Anacardiaceae | *Lannea* | ***welwitschii*** |
| Rubiaceae | *Massularia* | *acuminata* |
| Leguminosae | ***Millettia*** | *barteri, griffoniana, laurenti, manni, sanagana, versicolor* |
| Annonaceae | ***Monanthotaxis*** | *congolensis, diclina, klainii* |
| Annonaceae | *Monodora* | ***angolensis*** |
| Moraceae | *Musanga* | ***cecropioides*** |
| Moraceae | *Myrianthus* | ***arboreus*** |
| Lecythidaceae | *Napoleonaea* | ***vogelii*** |
| Rubiaceae | ***Nauclea*** | *didderichii, latifolia, pobeguinii, vanderguchtii* |
| Olacaceae | ***Ongokea*** | *gore* |
| Sapotaceae | *Pachystela* | ***brevipes*** |
| Leguminosae | ***Parkia*** | *bicolor, filicoidea* |
| Passifloraceae | *Paropsia* | *grewiodes* |
| Mimosaceae | *Pentaclethra* | *macrophylla* |
| Clusiaceae | *Pentadesma* | ***butyracea*** |
| Rubiaceae | *Polycoryne* | *fernandensis* |
| Rubiaceae | *Porterandia* | ***cladantha*** |
| Anacardiaceae | *Pseudospondias* | ***longifolia, microcarpa*** |
| Myrtaceae | *Psidium* | ***guineensis*** |
| Hypericaceae | *Psorospermum* | *febrifugum* |
| Rubiaceae | ***Psychotria*** | *penduncularis, venosa, voegliana* |
| Myristicaceae | *Pycnathus* | *angolensis* |
| Zingiberaceae | ***Renealmia*** | *cincinnata, macrocolea* |
| Humiriaceae | *Sacoglottis* | *gabonensis* |
| Burseraceae | *Santiria* | ***trimera*** |
| Flacourtiaceae | *Scottellia* | ***coriacea*** |
| Olacaceae | *Strombosiopsis* | ***tetrandra*** |
| Apocynaceae | ***Strychnos*** | *congolana, malacoclados* |
| Dilleniaceae | *Tetracera* | *podotricha* |
| Mimosaceae | *Tetrapleura* | *tetraptera* |
| Moraceae | *Treculia* | ***africana*** |
| Meliaceae | *Trichillia* | *prieureana* |
| Anacardiaceae | ***Trichoscypha*** | *abut, acuminata, anomala* |
| Euphorbiaceae | *Uapaca* | ***guinensis, heudelotii****, paludosa, togoensis, vanhouttei* |
| Annonaceae | *Uvaria* | ***versicolor****, scabrida, klaineana, psorosperma* |
| Annonaceae | *Uvariastrum* | ***pierreanum*** |
| Verbenaceae | *Vitex* | ***doniana*** |
| Annonaceae | *Xylopia* | *aethiopica, hypolampra, parviflora,* ***quintasii,*** *staudtii* |
| Cyperaceae | *Bulbostylis* | *densa, laniceps* |
| Cyperaceae | *Cyperus* | rotundus, sphacelatus |
| Cyperaceae | *Eleocharis* | variegata |
| Cyperaceae | *Fimbristylis* | pilosa |
| Cyperaceae | *Kyllinga* | echinata |
| Cyperaceae | *Rhynchospora* | corymbosa |
| Cyperaceae | *Scleria* | boivinii |
| Marantaceae | *Marantochloa* | cordifolia, filipes, purpurea |
| Marantaceae | *Megaphrynium* | macrostachyum, velutinum |
| Marantaceae | *Haumania* | liebrechtsiana |
| Marantaceae | *Hypselodelphys* | violacea |
| Marantaceae | *Sarcophrynium* | spp. |
| Marantaceae | *Trachyphrynium* | braunianum |
| Graminae |  | spp. |

**Table A2.** Summary of generalized additive mixed model examining the effect of Day of Year on fruit consumption by mandrills.

| GAM | | | | | |
| --- | --- | --- | --- | --- | --- |
| Component | Term | Estimate | Std Error | t | p |
| A. parametric coefficients | Intercept | 2.07 | 0.13 | 16.21 | <0.001 |
| Component | Term | **Effective Degrees of Freedom** | Reference. Degrees of Freedom | F | p |
| B. smooth terms | s(DayofYear) | 5.04 | 8.0 | 13.7 | <0.001 |
|  | s(DayofYear,Year) | 42.21 | 74.0 | 311.2 | <0.001 |
| Adjusted R^2^: 0.211 | | | | | |
| **MER** | | | | | |
| **Group** | **Estimate** | | **Std Error** | t | **p** |
| **Fixed Effects** | | | | | |
|  | Intercept | 2.07 | 0.13 | 15.8 | <0.001 |
| **Random Effect** | | | | | |
| Xr.0 | Standard Error s(DayofYear,Year) | 6.63 | | | |
| Xr.1 | Standard Error s(DayofYear,Year) | 1.92 | | | |
| Xr | Standard Error s(DayofYear) | 0.34 | | | |

**Table A3.** Summary of generalized additive model examining the effect of Day of Year on animal part consumption by mandrills.

| GAM | | | | | |
| --- | --- | --- | --- | --- | --- |
| Component | Term | Estimate | Std Error | t | p |
| A. parametric coefficients | Intercept | 1.39 | 0.14 | 10.11 | <0.001 |
| Component | Term | **Effective Degrees of Freedom** | Reference. Degrees of Freedom | F | p |
| B. smooth terms | s(DayofYear) | 5.37 | 8.0 | 11.91 | <0.001 |
|  | s(DayofYear,Year) | 37.42 | 74.0 | 385.75 | <0.001 |
| Adjusted R^2^: 0.147 | | | | | |
| **MER** | | | | | |
| **Group** | **Estimate** | | **Std Error** | t | **p** |
| **Fixed Effects** | | | | | |
|  | Intercept | 1.39 | 0.14 | 10.0 | <0.001 |
| **Random Effect** | | | | | |
| Xr.0 | Standard Error s(DayofYear,Year) | 3.51 | | | |
| Xr.1 | Standard Error s(DayofYear,Year) | 2.42 | | | |
| Xr | Standard Error s(DayofYear) | 0.23 | | | |

**Table A4.** Summary of generalized additive model examining the effect of Day of Year on dicot leaf consumption by mandrills.

| GAM | | | | | |
| --- | --- | --- | --- | --- | --- |
| Component | Term | Estimate | Std Error | t | p |
| A. parametric coefficients | Intercept | 0.02 | 0.11 | 0.14 | 0.8876 |
| Component | Term | **Effective Degrees of Freedom** | Reference. Degrees of Freedom | F | p |
| B. smooth terms | s(DayofYear) | 5.58 | 8.0 | 15.16 | <0.001 |
|  | s(DayofYear,Year) | 46.4 | 74.0 | 386.49 | <0.001 |
| Adjusted R^2^: 0.131 | | | | | |
| **MER** | | | | | |
| **Group** | **Estimate** | | **Std Error** | t | **p** |
| **Fixed Effects** | | | | | |
|  | Intercept | 0.02 | 0.11 | 0.14 | 0.8893 |
| **Random Effect** | | | | | |
| Xr.0 | Standard Error s(DayofYear,Year) | 5.34 | | | |
| Xr.1 | Standard Error s(DayofYear,Year) | 1.84 | | | |
| Xr | Standard Error s(DayofYear) | 0.30 | | | |

**Table A5.** Summary of generalized additive model examining the effect of Day of Year on other fiber consumption by mandrills.

| GAM | | | | | |
| --- | --- | --- | --- | --- | --- |
| Component | Term | Estimate | Std Error | t | p |
| A. parametric coefficients | Intercept | -0.166 | 0.194 | -0.855 | 0.3926 |
| Component | Term | **Effective Degrees of Freedom** | Reference. Degrees of Freedom | F | p |
| B. smooth terms | s(DayofYear) | 3.1 | 8.0 | 5.17 | <0.001 |
|  | s(DayofYear,Year) | 50.1 | 74.0 | 516.13 | <0.001 |
| Adjusted R^2^: 0.226 | | | | | |
| **MER** | | | | | |
| **Group** | **Estimate** | | **Std Error** | t | **p** |
| **Fixed Effects** | | | | | |
|  | Intercept | -0.166 | 0.195 | -0.853 | 0.3939 |
| **Random Effect** | | | | | |
| Xr.0 | Standard Error s(DayofYear,Year) | 6.328 | | | |
| Xr.1 | Standard Error s(DayofYear,Year) | 3.572 | | | |
| Xr | Standard Error s(DayofYear) | 0.175 | | | |

**Table A6.** Summary of generalized additive model examining the effect of Day of Year on crushed seed consumption by mandrills.

| GAM | | | | | |
| --- | --- | --- | --- | --- | --- |
| Component | Term | Estimate | Std Error | t | p |
| A. parametric coefficients | Intercept | -0.57 | 0.28 | -2.04 | 0.042 |
| Component | Term | **Effective Degrees of Freedom** | Reference. Degrees of Freedom | F | p |
| B. smooth terms | s(DayofYear) | 1.97 | 8.0 | 2.58 | <0.001 |
|  | s(DayofYear,Year) | 53.51 | 74.0 | 575.2 | <0.001 |
| Adjusted R^2^: 0.240 | | | | | |
| **MER** | | | | | |
| **Group** | **Estimate** | | **Std Error** | t | **p** |
| **Fixed Effects** | | | | | |
|  | Intercept | -0.57 | 0.30 | -2.04 | 0.042 |
| **Random Effect** | | | | | |
| Xr.0 | Standard Error s(DayofYear,Year) | 7.58 | | | |
| Xr.1 | Standard Error s(DayofYear,Year) | 5.26 | | | |
| Xr | Standard Error s(DayofYear) | 0.15 | | | |

**Table A7.** Summary of generalized additive model examining the effect of Day of Year on fungi consumption by mandrills.

| GAM | | | | | |
| --- | --- | --- | --- | --- | --- |
| Component | Term | Estimate | Std Error | t | p |
| A. parametric coefficients | Intercept | -2.62 | 0.26 | -9.96 | <0.001 |
| Component | Term | **Effective Degrees of Freedom** | Reference. Degrees of Freedom | F | p |
| B. smooth terms | s(DayofYear) | 0.0 | 8.0 | 0.0 | 1.0 |
|  | s(DayofYear,Year) | 41.0 | 74.0 | 223.66 | <0.001 |
| Adjusted R^2^: 0.07 | | | | | |
| **MER** | | | | | |
| **Group** | **Estimate** | | **Std Error** | t | **p** |
| **Fixed Effects** | | | | | |
|  | Intercept | -2.62 | 0.28 | -9.42 | <0.001 |
| **Random Effect** | | | | | |
| Xr.0 | Standard Error s(DayofYear,Year) | 5.30 | | | |
| Xr.1 | Standard Error s(DayofYear,Year) | 4.78 | | | |
| Xr | Standard Error s(DayofYear) | 0.0 | | | |

**Table A8.** Summary of generalized additive model examining the effect of Day of Year on monocot leaf consumption by mandrills.

| GAM | | | | | |
| --- | --- | --- | --- | --- | --- |
| Component | Term | Estimate | Std Error | t | p |
| A. parametric coefficients | Intercept | -2.69 | 0.41 | -6.6 | <0.001 |
| Component | Term | **Effective Degrees of Freedom** | Reference. Degrees of Freedom | F | p |
| B. smooth terms | s(DayofYear) | 3.86 | 8.0 | 10.08 | <0.001 |
|  | s(DayofYear,Year) | 27.95 | 74.0 | 221.27 | <0.001 |
| Adjusted R^2^: 0.097 | | | | | |
| **MER** | | | | | |
| **Group** | **Estimate** | | **Std Error** | t | **p** |
| **Fixed Effects** | | | | | |
|  | Intercept | -2.69 | 0.41 | -6.56 | <0.001 |
| **Random Effect** | | | | | |
| Xr.0 | Standard Error s(DayofYear,Year) | 2.46 | | | |
| Xr.1 | Standard Error s(DayofYear,Year) | 8.11 | | | |
| Xr | Standard Error s(DayofYear) | 0.15 | | | |

**Table A9.** Generalized additive model examining the relationship between day of year and overall diet breadth of mandrills.

| GAM | | | | | |
| --- | --- | --- | --- | --- | --- |
| Component | Term | Estimate | Std Error | t | p |
| A. parametric coefficients | Intercept | 1.16 | 0.02 | 62.46 | <0.001 |
| Component | Term | **Effective Degrees of Freedom** | Reference. Degrees of Freedom | F | p |
| B. smooth terms | s(DayofYear) | 3.96 | 8.0 | 9.22 | <0.001 |
|  | s(DayofYear,Year) | 25.23 | 74.0 | 113.23 | <0.001 |
| Adjusted R^2^: 0.128 | | | | | |
| **MER** | | | | | |
| **Group** | **Estimate** | | **Std Error** | t | **p** |
| **Fixed Effects** | | | | | |
|  | Intercept | 1.16 | 0.02 | 61.56 | <0.001 |
| **Random Effect** | | | | | |
| Xr.0 | Standard Error s(DayofYear,Year) | 0.31 | | | |
| Xr.1 | Standard Error s(DayofYear,Year) | 0.30 | | | |
| Xr | Standard Error s(DayofYear) | 0.02 | | | |

**Table A10.** Generalized additive model examining the relationship between day of year and breadth of fruit species consumption by mandrills.

| GAM | | | | | |
| --- | --- | --- | --- | --- | --- |
| Component | Term | Estimate | Std Error | t | p |
| A. parametric coefficients | Intercept | 0.38 | 0.05 | 6.89 | <0.001 |
| Component | Term | **Effective Degrees of Freedom** | Reference. Degrees of Freedom | F | p |
| B. smooth terms | s(DayofYear) | 5.49 | 8.0 | 20.07 | <0.001 |
|  | s(DayofYear,Year) | 42.41 | 74.0 | 256.42 | <0.001 |
| Adjusted R^2^: 0.203 | | | | | |
| **MER** | | | | | |
| **Group** | **Estimate** | | **Std Error** | t | **p** |
| **Fixed Effects** | | | | | |
|  | Intercept | 0.38 | 0.06 | 6.81 | <0.001 |
| **Random Effect** | | | | | |
| Xr.0 | Standard Error s(DayofYear,Year) | 1.51 | | | |
| Xr.1 | Standard Error s(DayofYear,Year) | 0.98 | | | |
| Xr | Standard Error s(DayofYear) | 0.09 | | | |

**Table A11.** Results of chi-squared tests examining differences in binomial presence of mandrill food types in fecal samples, when fruit is present or absent in the sample.

| **Type** | **Fruit** | **Proportion** | **CI Lower Bound** | **CI Upper Bound** | ***p*** |
| --- | --- | --- | --- | --- | --- |
| **Animal Parts** | **Present** | 0.74 | 0.72 | 0.75 | <0.001 |
|  | **Absent** | 0.82 | 0.79 | 0.85 |  |
| **Dicot Leaves** | **Present** | 0.49 | 0.48 | 0.51 | <0.001 |
|  | **Absent** | 0.61 | 0.57 | 0.64 |  |
| **Other** | **Present** | 0.44 | 0.42 | 0.45 | <0.001 |
|  | **Absent** | 0.85 | 0.82 | 0.88 |  |
| **Crushed Seeds** | **Present** | 0.40 | 0.38 | 0.41 | <0.001 |
|  | **Absent** | 0.57 | 0.53 | 0.61 |  |
| **Fungi** | **Present** | 0.12 | 0.1 | 0.13 | <0.41 |
|  | **Absent** | 0.10 | 0.08 | 0.13 |  |
| **Monocot Leaves** | **Present** | 0.09 | 0.08 | 0.1 | <0.6 |
|  | **Absent** | 0.10 | 0.08 | 0.13 |  |

**Table A12.** Summary of generalized linear mixed model examining the relationship between fruit availability and consumption by mandrills, with random slopes for each tree genus.

| **Estimate** | | | **Standard Error** | **Statistic** | ***p*** |
| --- | --- | --- | --- | --- | --- |
| **Fixed effects** | | | | | |
|  | Intercept | -5.377 | 0.363 | -14.801 | <0.001 |
|  | Scaled Fruit Availability Score | 2.364 | 1.140 | 2.074 | 0.038 |
| **Random effects** | | | | | |
| Genus | Standard Deviation: Intercept | 1.915 | | | |
| Genus | Standard Deviation: Scaled Fruit Availability Score | 9.648 | | | |
| Square root of the estimated residual variance: 1.0 | | | | | |
